# Dynamic interactions and Ca^2+^-binding modulate the holdase-type chaperone activity of S100B preventing tau aggregation and seeding

**DOI:** 10.1038/s41467-021-26584-2

**Published:** 2021-11-01

**Authors:** Guilherme G. Moreira, François-Xavier Cantrelle, Andrea Quezada, Filipa S. Carvalho, Joana S. Cristóvão, Urmi Sengupta, Nicha Puangmalai, Ana P. Carapeto, Mário S. Rodrigues, Isabel Cardoso, Güenter Fritz, Federico Herrera, Rakez Kayed, Isabelle Landrieu, Cláudio M. Gomes

**Affiliations:** 1 Biosystems & Integrative Sciences Institute, Faculdade de Ciências, Universidade de Lisboa, Lisboa, Portugal; 2 Departamento de Química e Bioquímica, Faculdade de Ciências, Universidade de Lisboa, Lisboa, Portugal; 3 CNRS ERL9002 Integrative Structural Biology, F-59000 Lille, France; 4 Univ. Lille, Inserm, CHU Lille, Institut Pasteur de Lille, U1167 - RID-AGE - Risk Factors and Molecular Determinants of Aging-Related Diseases, F-59000 Lille, France; 5 Mitchell Center for Neurodegenerative Diseases, University of Texas Medical Branch, 301 University Blvd, Medical Research Building, Room 10.138C, Galveston, TX 77555-1045 USA; 6 Departments of Neurology, Neuroscience and Cell Biology, University of Texas Medical Branch, Galveston, TX USA; 7 Departamento de Física, Faculdade de Ciências, Universidade de Lisboa, Lisboa, Portugal; 8 i3S—Instituto de Investigação e Inovação em Saúde, Universidade do Porto, Porto, Portugal; 9 IBMC - Instituto de Biologia Molecular e Celular, Universidade do Porto, Porto, Portugal; 10 Instituto de Ciências Biomédicas Abel Salazar (ICBAS), 4050-013 Porto, Portugal; 11 Institute of Biology, Department of Cellular Microbiology, University of Hohenheim, Stuttgart, 70599 Germany

**Keywords:** Biophysical chemistry, Protein folding, Structural biology

## Abstract

The microtubule-associated protein tau is implicated in the formation of oligomers and fibrillar aggregates that evade proteostasis control and spread from cell-to-cell. Tau pathology is accompanied by sustained neuroinflammation and, while the release of alarmin mediators aggravates disease at late stages, early inflammatory responses encompass protective functions. This is the case of the Ca^2+^-binding S100B protein, an astrocytic alarmin which is augmented in AD and which has been recently implicated as a proteostasis regulator, acting over amyloid β aggregation. Here we report the activity of S100B as a suppressor of tau aggregation and seeding, operating at sub-stoichiometric conditions. We show that S100B interacts with tau in living cells even in microtubule-destabilizing conditions. Structural analysis revealed that tau undergoes dynamic interactions with S100B, in a Ca^2+^-dependent manner, notably with the aggregation prone repeat segments at the microtubule binding regions. This interaction involves contacts of tau with a cleft formed at the interface of the S100B dimer. Kinetic and mechanistic analysis revealed that S100B inhibits the aggregation of both full-length tau and of the microtubule binding domain, and that this proceeds through effects over primary and secondary nucleation, as confirmed by seeding assays and direct observation of S100B binding to tau oligomers and fibrils. In agreement with a role as an extracellular chaperone and its accumulation near tau positive inclusions, we show that S100B blocks proteopathic tau seeding. Together, our findings establish tau as a client of the S100B chaperone, providing evidence for neuro-protective functions of this inflammatory mediator across different tauopathies.

## Abstract (web summary)

The calcium binding protein S100B is an abundantly expressed protein in the brain and has neuro-protective functions by inhibiting Aβ aggregation and metal ion toxicity. Here, the authors combine cell biology and biochemical experiments with chemical kinetics and NMR measurements and show that S100B protein is an extracellular Tau chaperone and further characterize the interactions between S100B and Tau.

## Introduction

Protein aggregation is a hallmark in several neurodegenerative diseases, which are commonly characterized by disrupted proteostasis and massive accumulation of misfolded proteins that overload the chaperone quality-control system and form neurotoxic inclusions^1,2^. Among these is tau, an intrinsically disordered protein (IDP) associated with microtubule (MT) stabilization^3–5^, whose deposition is implicated in multiple neurodegenerative tauopathies, including frontotemporal dementia and parkinsonism linked to chromosome 17 (FTDP-17) and Alzheimer’s disease (AD)^6^. In AD, disruption in tau binding to MT destabilizes the latter, a phenomenon that is aggravated by abnormal hyperphosphorylation^7^. Released tau then aggregates into paired helical filaments (PHF) that form neurofibrillary tangles (NFT)^8^.

Neuroinflammation is another cardinal feature commonly observed in neurodegenerative conditions^9,10^. In AD, early inflammation during the prodromal stage is recognized to involve important molecular changes in brain cells, which are relevant to AD development^11^. The accumulation of protein aggregates in the brain results in a rise in inflammatory mediators at early disease stages, that involves increased expression and release of several alarmins, including S100B^12^. S100B is a member of the Ca^2+^-binding S100 family of proteins and is produced primarily by astrocytes^13^. It occurs constitutively at high levels in the brain (estimated 0.5% of brain protein^14^) and gets further increased with aging, in traumatic brain injury and AD^15–17^. Interestingly, the expression and abundance of S100 proteins correlates with disease progression in AD mice models^16^. S100B acts both intra- and extracellularly and its expression levels dictate its location. Secreted S100B is not toxic and is taken up by neurons^18^. Similarly to other multitasking alarmins^19^, S100B is a multifunctional protein implicated in the regulation of proliferation, differentiation, apoptosis, Ca^2+^ homeostasis, energy metabolism, and inflammation^20^. In AD, extracellular S100B is present at high-levels nearby plaques and this led to the discovery of its chaperone function^16^. We recently uncovered that S100B physically interacts with Aβ42 in a Ca^2+^-dependent manner, delaying its aggregation and preventing neurotoxicity^21^. This finding unveiled a regulatory mechanism with potential relevance in AD, through which S100B acts as a chaperone that counteracts Aβ aggregation and toxicity of amyloid at early stages. Increasing Aβ proteotoxicity at late disease stages abolishes this protective role and the pro-inflammatory activity of S100B takes over with disease-aggravating consequences^11,16^.

Interestingly, S100B has been previously recognized as a MT-regulating factor through uncharacterized mechanisms and interactions with tau, whose details remain however elusive^22–25^. Such earlier reports provided the first indications for a Ca^2+^-dependent inhibitory action of S100B on MT assembly, presumed to involve binding between tau and S100B^25^, which was later demonstrated from covalent binding studies^26^. This prompted us to postulate that tau might also be a client for the S100B chaperone. The investigations reported here establish that tau physically interacts with S100B at a binding cleft that we previously associated with chaperone activity toward Aβ, as determined by nuclear magnetic resonance spectroscopy (NMR)^21^ and confirmed by SAXS. A dynamic interaction between S100B and tau was also established in living cells, as observed by bimolecular fluorescence complementation (BiFC) assays. Using aggregation-kinetics experiments and mechanistic modeling analysis, we showed that the fly casting-type interactions^27^ between the two proteins result in a delay of tau aggregation. Importantly, we uncover that S100B is an effective chaperone at low substoichiometric levels and that S100B also mitigates the seeding potency of tau oligomers, as determined using a cell reporter system. Altogether, this study presents a mechanism with implications in AD and other tauopathies, implicating S100B as an extracellular holdase-type chaperone that mitigates tau aggregation and seeding.

## Results

### Dynamics of tau and S100B interaction in destabilized microtubules in living cells

**Fig. 1:**
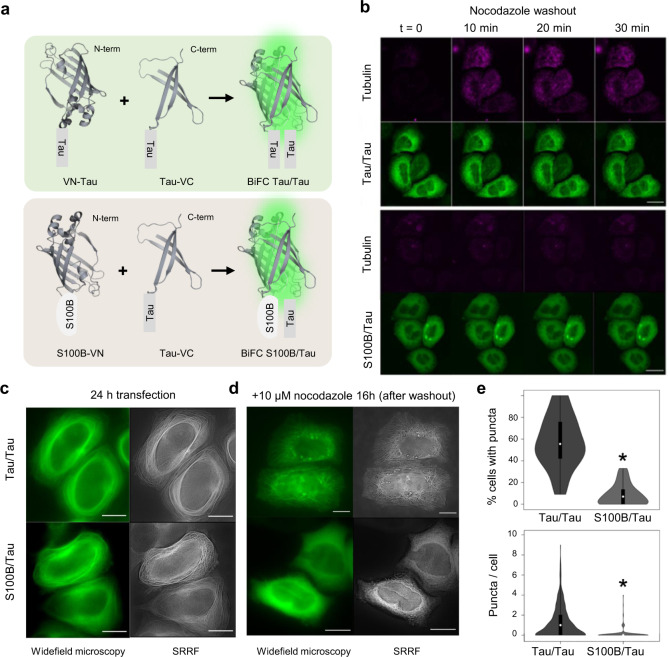
S100B and tau colocalize in living cells and interactions persist upon microtubule destabilization. **a** Representation of N- and C-terminal halves of Venus constructs fused to tau and S100B termini and gain of fluorescence based on bimolecular fluorescence complementation. **b** HeLa cells were transfected with the BiFC constructs tau/tau and S100B/tau and incubated with 10 µM of nocodazole for 16 h. Nocodazole was then removed, and cells were visualized immediately after washing out nocodazole (*t* = 0) in time lapse for 30 min. Scale bar: 20 µm. **c**, **d** Wide-field microscopy and super-resolution radial fluctuations (SRRF) reconstruction of HeLa cells (**c**) after 24 h of transient transfection with the BiFC constructs tau/tau and S100B/tau; and (**d**) after 16 h of incubation with 10 µM of nocodazole, imaged ~15 min after washout. Scale bar: 20 µm. **e** Violin-plot representation of cells forming puncta (top panel) and number of puncta per cell (bottom panel) after 16 h of incubation with 10 µM of nocodazole. Plots are presented with floating boxes showing mean (white dot) and 1st and 3rd quartiles (floating black boxes). Minimum and maximum values of the distribution are shown as the bottom and top of the violin plot. The number of cells analyzed was 927 (tau/tau) and 873 (tau/S100B) in a total of 65 pictures/group from 4 independent experiments. Data were analyzed by means of a two-tailed *t*–student test. *, significant versus tau/tau, top *p* = 0.0097 and bottom *p* = 3.6673 × 10^−34^.

Following earlier evidence for an interaction between tau and S100B obtained from co-elution in affinity-chromatography experiments using brain-derived proteins^22–25^, we here set to investigate the interaction between tau and S100B in living cells. For this, we resorted to bimolecular fluorescence complementation (BiFC) assays, an approach previously validated to image the interaction of tau with the microtubule (MT)-network proteins in mammalian cells^28,29^. Cells were cotransfected with the tau-VC and S100B-VN BiFC pair of constructs to visualize the interaction between these two proteins, while a tau–tau BiFC system was used as control (Fig. 1a). When the proteins interact, the two nonfluorescent Venus fragments are brought together and reconstitute the fluorescence. We observed that tau and S100B interact with each other and are attached to the MT network, as shown by wide-field fluorescence microscopy and super-resolution radial fluctuation (SRRF) analysis^30^ (Fig. 1c, d and Supplementary Fig. 1). The interaction between S100B and tau was further confirmed by colocalization analysis in live and fixed cells in experimental setups not dependent on BiFC, as demonstrated by experiments using HeLa cells, U-251 MG glioblastoma cells, and differentiated SH-SY5Y neuroblastoma cells (Supplementary Fig. 2). In all cases, the average colocalization levels of S100B and tau were positive, indicating that the S100B and tau interaction occurs independently of BiFC, and that it is likely transient and dependent on the biological context.

Under the tested experimental conditions in the BiFC experiments, there is no apparent MT depolymerization. Nevertheless, the morphology of the cells presents discrete differences: a more intricate network is observed in S100B/tau than in tau/tau, which is located mainly in the periphery of the cell (Fig. 1c). To investigate this aspect, we further explored the S100B/tau interaction in the presence of nocodazole, a drug that binds β-tubulin and interferes with MT polymerization and dynamics^31^. Incubation of cells transfected with the BiFC tau/tau constructs with 10 µM nocodazole for 16 h indeed disrupted the MT network (Fig. 1d), resulting in the disintegration of MT and the formation of puncta that are observed in approximately 60% of the cells (Fig. 1e). On the other hand, the same experiment performed in cells expressing the BiFC S100B/tau constructs results in more drastic disintegration of the MT upon nocodazole destabilization (Fig. 1d), with diffuse fluorescence observed in the cell cytoplasm and only residual puncta in 8% of the cells (Fig. 1e). Control-immunoblot analysis of tau expression showed that levels of expression are similar when cells are transfected with tau/tau and S100B/tau, both in the presence and absence of nocodazole, thus ruling out the effects of protein levels on the observed phenomena (Supplementary Fig. 3)

This is compatible with an additive effect of nocodazole and S100B on MT destabilization, and with the reported interaction between S100B and tubulin^32^. We then posited that the interaction between S100B and tau in the destabilized MT would also influence reassembly. To test this hypothesis, we carried out nocodazole-washout experiments and observed that, in cells expressing the BiFC tau/tau constructs, the small puncta disappear as tau attaches to the forming MT as evidenced from the colocalization of the two signals in the time lapse (Fig. 1b). The fact that puncta are dynamic and quickly dispel when the MT network is restored, might be an indicator of their liquid-like nature^33^. On the other hand, in cells expressing BiFC S100B/tau constructs, the microtubule network does not polymerize again after removing nocodazole in the presence of S100B, even after one hour (Fig. 1b). Altogether, these experiments in live cells using nocodazole perturbation show that S100B colocalizes with tau in MT, and that this interaction persists upon MT destabilization.

### S100B binds full-length and tau fragments in a Ca^2+^-dependent manner

**Fig. 2:**
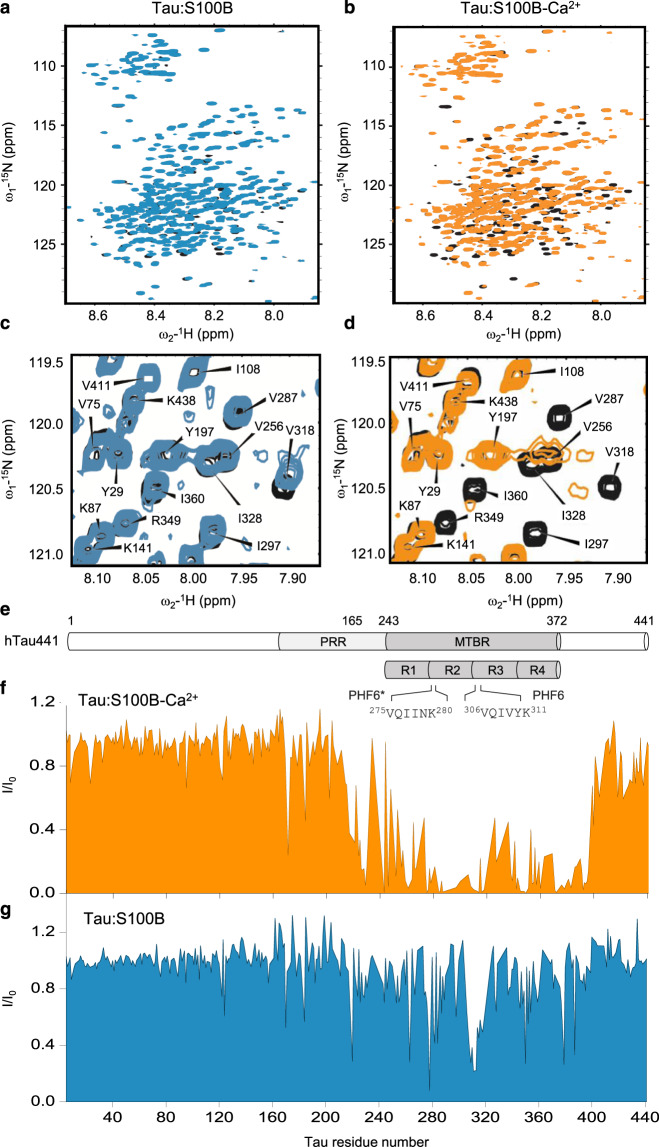
Mapping S100B interactions to tau. **a**, **b** 2D ^1^H,^15^N HSQC spectra of 100 µM ^15^N-tau alone (black), in the presence of 120 µM S100B and 1.25 mM EDTA (**a**, blue), or in the presence of 120 µM S100B and 250 µM CaCl_2_ (**b**, orange). **c**, **d** Detail of HSQC-NMR spectra evidencing tau-resonance perturbations corresponding to residues affected by S100B binding. Parts per million (ppm). **e** Schematic representation of full-length tau highlighting the PRR and MTBR as well as the repeat regions R1–R4 and the aggregation-prone segments PHF6* and PHF6, aligned with the x axis of plots **f** and **g**. **f**, **g** Intensity variation of each tau resonance in the presence of S100B and in the presence (**f**, orange) or the absence (**g**, blue) of calcium. Ratio of peak intensity over initial peak intensity (I/I_0_).

To investigate the structural details of the S100B:tau interaction, we resorted to solution nuclear magnetic resonance (NMR) spectroscopy, which allows mapping of residues and regions affected by the physical interaction between S100B and tau (Fig. 2). As a first step, ^1^H,^15^N HSQC 2D spectrum of full-length ^15^N-labeled tau (hTau441) was recorded in the presence or absence of S100B, either with calcium or EDTA for both conditions. Comparison of the spectra obtained with EDTA for ^15^N-tau alone with that of ^15^N-tau in the presence of S100B at a molar ratio of 1:1.2 showed nearly no difference of chemical shift value or intensity of the tau resonances upon addition of S100B (Fig. 2a, c).

However, when the experiment was done with Ca^2+^-bound S100B (S100B-Ca^2+^), as obtained by adding Ca^2+^ instead of EDTA to the solution, this resulted in numerous perturbations of the resonances in the ^15^N-tau 2D NMR spectrum, with multiple resonances broadened beyond detection (Fig. 2b, d). Indeed, Ca^2+^ binding to S100 proteins is known to promote conformational changes that expose a broad hydrophobic surface that enables interactions with a variety of target proteins^34,35^. Therefore, in the apo state, such interaction-prone surfaces on S100B are minimal and this explains the discrete perturbations observed in the spectrum of tau obtained in the presence of apo S100B (Fig. 2g).

A control experiment showed that Ca^2+^ had no effect on tau resonances (Supplementary Fig. 4), indicating that the observed changes result from binding of ^15^N-tau to S100B-Ca^2+^. As the assignment of most tau resonances is available, we can link these intensity perturbations of resonances to specific amino acid residues in the tau sequence (Fig. 2e). Indeed, comparison of the intensity ratio of the corresponding resonances (I/I_0_) in the spectrum of ^15^N-tau in the presence of S100B-Ca^2+^ (I) relative to the intensity in the spectrum of tau alone in solution (I_0_) allowed mapping the interaction region (Fig. 2f, g). Perturbations observed as loss of resonance intensity corresponded to amino acid residues located within the proline-rich region (PRR) and the microtubule-binding region (MTBR) of tau, which harbors repeat domains R1–R4 (Fig. 2e). This experiment showed that S100B interacts with full-length tau in a strict Ca^2+^-dependent manner, and that the interaction region is large and affects both the PRR and the MTBR. Given that these regions span about 200 amino acids altogether, the data suggest that several S100B-binding sites are present along the tau sequence. This large region of interaction comprises the key binding sites within the MTBR domain that also interact with several classical chaperones such as Hsp90 and DnaJ proteins, which inhibit tau aggregation^36^.

### Tau binds preferentially at the S100B dimer interfacial cleft

**Fig. 3:**
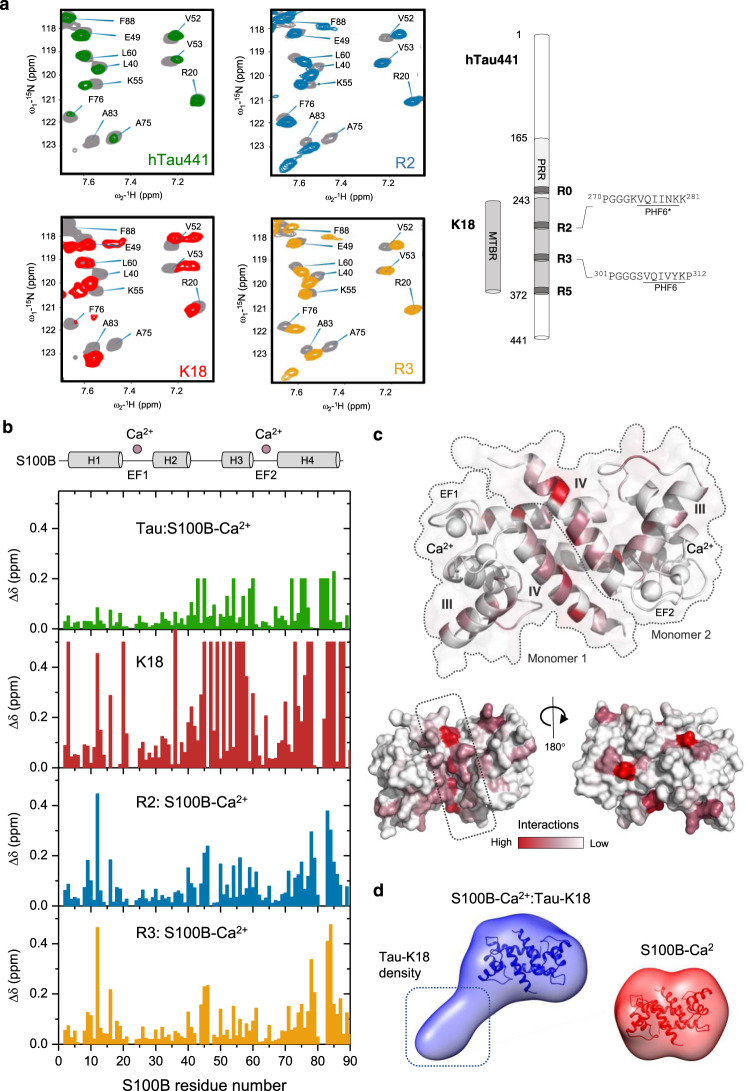
Mapping tau interactions to S100B. **a** Zoom of HSQC spectra evidencing chemical shift perturbation of 100 µM ^15^N-S100B residues in the presence of 1.25 mM CaCl_2_ and 160 µM full-length tau (green), 100 µM K18 (or MTBR, red), 250 µM of R2 (blue) and R3 peptide (yellow). Spectra changes upon binding of tau (green), K18 (red), and R2 (blue) and R3 (yellow) peptides to S100B-Ca^2+^ (alone in gray). **b** Plots of the chemical shift perturbation along S100B sequence following addition (from top to bottom) of full-length tau (molar ratio 1:1.6), tau K18 fragment (molar ratio 1:1), and tau peptides (molar ratio 1:2.5). Peaks broadened beyond detection are represented with a fixed arbitrary value of 0.2 (with tau) and 0.5 (with K18). Parts per million (ppm). A scheme of the secondary structures of S100B is presented above the plots, scaled according to the sequence of the *x* axis. **c** 3D mapping of the R2:S100B-Ca^2+^ spectral perturbations identified the S100B dimer interfacial clef (dotted box) involving helix IV as the major attachment point, chemical shift perturbations (as in **a**) are color-coded on the protein surface [Protein Data Bank (PDB) code: 2H61]. See Methods for details, Supplementary Figs 7 and 9 and Supplementary Movie 1. **d** Ab initio SAXS-derived envelopes of S100B-Ca^2+^:K18 complex (blue) and S100B-Ca^2+^ (red), see Supplementary Table 1 for data-collection and scattering-derived parameters. The crystal structure of S100B-Ca^2+^ fitted to both molecular envelopes is shown in cartoon representation.

To establish which S100B regions are involved in interactions with tau, the reverse NMR experiment was performed using ^15^N-S100B-Ca^2+^. The S100B assignment used for the interaction-mapping experiment was based on previously published data^21^, transferred to our experimental conditions by temperature titration (Supplementary Fig. 5). Modification of the chemical shift values for all the resonances of S100B-Ca^2+^, compared with S100B, evidenced a global conformational rearrangement. This observation is in agreement with reports that Ca^2+^ binding results in exposure of a large hydrophobic surface and widening of the dimer interface involving changes in helices III and IV orientation^34,35^ (Supplementary Fig. 6). For the reverse-titration experiments, we employed full-length tau and two fragments from each of the identified interacting regions, namely the F5 (residues 165–245) for the PRR and the K18 fragment (residues 244–372) for the MTBR, as well as peptides R0, R2, R3, and R5 (further discussed below) from these regions. All these proteins and peptides were added to ^15^N-labeled S100B, and the corresponding spectra were recorded, with no precipitation occurring during these experiments in any of the tested conditions (Fig. 3a and Supplementary Fig. 7). The observed chemical shift perturbations were used to map the interaction sites on the S100B protein structure (Fig. 3b and Supplementary Fig. 8). Major perturbations are observed along helix IV at the S100B dimer interface, that define a binding cleft^35^ that is also the binding region for the Aβ peptide^21^, as well as in orthogonal helix III (Fig. 3c and Supplementary Fig. 9). We next carried out paramagnetic relaxation enhancement (PRE) experiments, which report spatial proximity of S100B residues to probes attached to K18, to obtain additional evidence that the interaction of tau takes place within this surface region of S100B-Ca^2+^. Conversely to NMR binding-site mapping, PRE experiments distinguish between perturbations due to direct interaction and indirect conformational change due to the binding. The comparison of the intensity profile of resonances in the NMR spectra of ^15^N-S100B-Ca^2+^, in the presence of spin-labeled K18 normalized on its diamagnetic counterpart with a reduced nitroxide, confirmed that the interaction takes place at the surface that exposes the binding cleft formed by helix IV in the S100B dimer. The first 50 residues were indeed globally unaffected by the paramagnetic probe, except for residues Val8, Leu10, and Phe14 located in helix I, at the bottom of the cleft. This observation agrees with the large chemical shift perturbation observed for residue Asp12. Resonances from residues of helices III and IV are already broadened due to K18 interaction itself and could not be used in the analysis. Decrease of the intensities of the resonances for residues 60–70, located in the connecting loop between helices III and IV, reaches about 50% of their maximum intensity in the absence of the probe, confirming the proximity of K18 with this loop region in S100B (Supplementary Fig. 10).

In addition, to further investigate possible conformational changes in S100B upon binding of tau domains, we resorted to far-ultraviolet circular dichroism (far UV-CD) to monitor secondary structure changes during titration experiments. The far UV-CD spectrum of S100B, which is typical of a well-defined α-helical fold, has not changed upon titration with K18, ruling out substantial structural rearrangements resulting from interactions with tau (Supplementary Fig. 11).

We further characterized the interaction between S100B and K18 using small angle X-ray scattering (SAXS). SEC-coupled SAXS data were obtained for the S100B homodimer, S100B dimer mixed in equimolar amounts with K18 or K18 alone as a control. The results obtained for K18 are in good agreement with previous SAXS studies on tau^37,38^ revealing a flexible protein. Data analysis and 3D reconstruction of the S100B–K18 complex using different approaches yielded very similar molecular envelopes with a volume correlation of 0.93 (Fig. 3d and Supplementary Fig. 12).

SAXS data also show that S100B remains folded in the dimer state upon interaction with tau. The reconstructed particle clearly shows a density corresponding to one S100B dimer and an additional electron density accounting for about 16 kDa (i.e., K18) bound to S100B-Ca^2+^, and is predominantly indicative of a 1:1-interaction stoichiometry. The vast part of the additional density is protruding on one side of the S100B dimer, although some additional density is also observed in the region corresponding to the dimer interface. This reveals that, while parts of tau interact with the S100B surface, some of its regions remain flexible (Fig. 3d).

### S100B binds preferentially repeat regions in tau MTBR

The reverse NMR-titration experiments confirmed that both fragments from the PRR (F5 fragment) and the MTBR (K18 fragment) can bind independently to S100B (Fig. 3 and Supplementary Fig. 9), supporting the evidence for multiple dynamic binding sites, similarly to what was observed for the interaction between Aβ42 and S100B^21^. To evaluate this aspect, we estimated the binding affinity between S100B-Ca^2+^ and hTau441 and the K18 fragment. For that, we performed ANS-fluorescence-monitored binding experiments as the interaction of tau with S100B-Ca^2+^ promotes changes in the emission spectrum of ANS bound to S100B-Ca^2+^, which has allowed to estimate apparent dissociation constants in the 0.2–0.7 μM range (Supplementary Fig. 13). The similar apparent binding affinities determined for the two interaction pairs suggest that K18 recapitulates the interaction between hTau441 and S100B, also as observed by NMR, and therefore likely corresponds to the main interacting region.

To further narrow the tau regions that preferentially interact with S100B, we resorted to four 12-residue-long tau peptides for additional NMR analysis (Fig. 3a). Two of the peptides (peptides R2 and R3) correspond to segments within the K18 region, each one comprising respectively the PHF6* and PHF6 amyloidogenic segments. As the interaction region extends beyond the MTBR, into the PRR and C-terminal regions, we also selected peptides R0 and R5 from these regions. All peptides were water-soluble and addition of each of the peptides to ^15^N-S100B led to chemical shift perturbations that were more notorious for peptides R2 and R3 (Fig. 3 and Supplementary Fig. 7 and 8). Mapping of the perturbations on the surface of S100B structure identified residues in the cleft as the primary interactions, with the corresponding resonances experiencing the largest chemical shift value changes. Chemical shift perturbations were observed for the perpendicular helix III, which might be due partly to a change in orientation upon binding (Fig. 3c and Supplementary Movie 1). The extent of perturbations observed in the S100B spectra upon addition of tau peptides was lower compared with those recorded upon addition of the larger tau fragments, as less resonances were affected. This might be due to the smaller size of the peptides but could also indicate that the tau fragments establish additional charge-driven interactions with the S100B surface, similarly to what has been proposed for Aβ42^39^. To further characterize the interaction between the tau peptides and S100B-Ca^2+^, we carried out NMR-monitored titration experiments. 2D spectra of ^15^N-S100B-Ca^2+^ were acquired for each titration point, corresponding to increasing amounts of tau peptides (Supplementary Fig. 14). Comparison of the spectral series for each peptide showed gradual perturbation of the resonances, which sample the bound and free conformations on a fast timescale. As the variation of the chemical shift is proportional to the quantity of formed complex, the dissociation constants (K_d_) can be calculated for each peptide by fitting to a saturation curve (Supplementary Fig. 14b). We determined K_d_ of 519 ± 202 µM for R0, 163 ± 39 µM for R2, and 266 ± 39 µM for R3, showing that S100B can bind multiple peptides along tau sequences. In addition, the direction of the S100B resonance shifts was similar for all the added peptides, as well as for tau and tau fragments, indicating similar binding modes for all cases (Supplementary Fig. 14c). These weak interactions, in the hundred µM range, suggest that S100B can switch between these sites to form a dynamic complex with tau. The higher affinity that characterizes the complex with the K18 fragment is likely due to an avidity effect, resulting from the establishment of these multiple weak interactions. Therefore, although S100B can bind a range of peptides with various sequences, which are nevertheless all characterized by the presence of basic and hydrophobic residues, the interaction regions in tau can be narrowed predominantly to the PHF6*/PHF6-containing segments within the MTBR, and in S100B to the dimer interfacial binding cleft, which had been previously described as a preferential interaction site for monomeric Aβ42^21,39^.

### S100B is a potent Ca^2+^-dependent inhibitor of tau aggregation

**Fig. 4:**
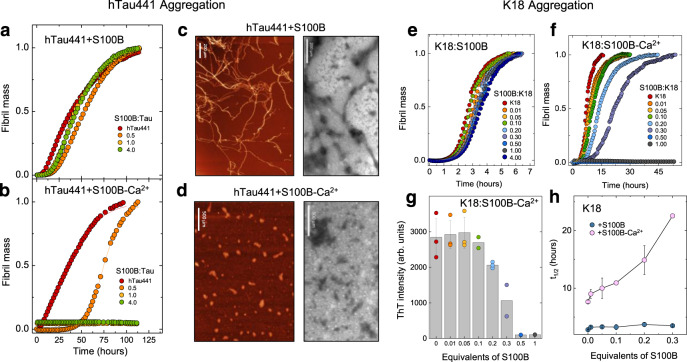
S100B-Ca^2+^ inhibits full-length tau and K18-fragment aggregation. **a**, **b** Fibril formation of 25 µM hTau441 (red) in 50 mM Tris, pH 7.4, in the absence (**a**) or the presence (**b**) of 1.1 mM CaCl_2_ at 37 °C under agitation in the presence of 0.5 (orange), 1.0 (yellow), and 4.0 (green) molar equivalents of S100B. Aggregation induced by 0.5 mg/mL heparin addition. **c**, **d** AFM (left) and TEM (right) imaging of end-time species of hTau441 aggregation in the absence (**c**) or the presence (**d**) of Ca^2+^ at 10 µM tau and at a S100B:tau molar ratio of 1. Scale bars, 200 µm (**c**) and 500 µm (**d**). Independent experiments of AFM and TEM morphological analysis were performed at least three times. **e**, **f** Fibril formation of 10 µM K18 (red) in 50 mM Tris, pH 7.4, in the absence (**e**) or the presence (**f**) of 1.1 mM CaCl_2_ at 37 °C under agitation in the presence of 0.01 (orange), 0.05 (yellow), 0.1 (green), 0.2 (light blue), 0.3 (purple), 0.5 (blue), 1.0 (gray), and 4.0 (dark blue) molar equivalents of S100B. Aggregation induced by 90 µg/mL heparin addition. Plots represent averaged normalized intensity curves obtained from three independent replicates for each of the tested conditions. **g** ThT intensity in arbitrary units (arb. units) of end-time point K18 aggregation in the presence of 1.1 mM CaCl_2_ (same color code); **h** half-time of K18 aggregation in the absence (blue) or the presence (light pink) of 1.1 mM CaCl_2_. Data are presented as mean values ± SD from *n* = 3 independent experiments except for samples with 0.1 and 0.3 equivalents of S100B for which *n* = 2. See Methods for details.

We then tested if S100B acts as an inhibitor of tau aggregation, considering its binding to the MTBR domain and repeat regions, which include the two hexapeptide motifs (PHF6*- 275–VQIINK-280 and PHF6-306–VQIVYK-311) responsible for aggregation of tau into filaments^40,41^. We carried out ThT-monitored aggregation kinetics of full-length tau using the well-established heparin-fibrillation assay^42,43^. We observed the typical sigmoid tau aggregation curve and noted that when apo-S100B is included in the assay, at up to a S100B:tau molar ratio of 4, the aggregation kinetics is not affected (Fig. 4a). However, when the same experiment is carried out in the presence of S100B-Ca^2+^, a pronounced inhibition is observed: at a molar ratio of 0.5, the reaction half-time (t_1/2_) increased from 10.7 to 55.4 h, and at stoichiometric conditions, a complete inhibition of tau aggregation is observed, with no fibril formation at up to 150 h of reaction (Fig. 4b). Appropriate controls revealed that the inhibition of tau aggregation by S100B is independent of the employed amyloid fluorophore probe and aggregation inducer (Supplementary Fig. 15) and that Ca^2+^ has a nearly negligible effect on tau-aggregation rate, and that neither S100B nor S100B-Ca^2+^ form ThT-positive species under the tested experimental conditions, in agreement with previous reports^44^.

The inhibitory effect of S100B-Ca^2+^ over tau fibrillation was also confirmed by atomic force microscopy (AFM) and transmission electron microscopy (TEM), which showed that no tau fibrils are formed in the presence of S100B-Ca^2+^ at the reaction endpoint. We rather observed the formation of diverse oligomeric species, presumably comprising tau:S100B complexes, as well as sparse short tau protofibrils (Fig. 4c, d). We carried out similar kinetic experiments using the K18 tau fragment from the MTBR as model, which comprises the PHF6*/PHF6 hexapeptide motifs. As for hTau441, addition of apo-S100B has a negligible effect on the aggregation rate of K18 (Fig. 4e), while S100B-Ca^2+^ results in a more pronounced inhibitory effect at much lower K18:S100B molar ratios (Fig. 4f). The reaction is completely abolished at a molar ratio of K18:S100B = 0.5 and above; the half-time of the aggregation reaction increases sharply, and the ThT intensity is practically abolished (Fig. 4g, h).

We then investigated how S100B influences tau aggregation using K18 as a model. Single-molecule studies have shown that the K18 tau fragment undergoes aggregation through a nucleation–conversion–polymerization mechanism^45^. Our assays suggest that tau K18 aggregation is well described by global analysis using a model involving secondary nucleation processes, given the linear dependence of t_1/2_ on the initial concentration of monomeric tau and the scaling exponent *γ* = −0.68, which is comparable to that obtained for other systems that follow this mechanism^21,46^. We observed that S100B-Ca^2+^ at a molar ratio S100B:K18 = 0.3 did not affect the dominant mechanism of tau aggregation as scaling remains linear. However, the dependencies of the relative combined rate constants obtained from the fits of K18 aggregation at variable S100B-Ca^2+^ concentrations showed a stronger effect of S100B on secondary nucleation (${k}_{+}{k}_{2}$) rather than on primary pathways (${k}_{n}{k}_{+}$) (Supplementary Fig. 16). Previous work established that S100B-Ca^2+^ also inhibits the aggregation of Aβ42 through interactions established at the exposed interfacial cleft^21^, implying the possibility for a crossed regulatory action of S100B and these two aggregation-prone proteins. To gain some initial insights into this process, we tested the inhibitory effect of S100B-Ca^2+^ over K18 aggregation in the presence of Aβ42. The results obtained suggest a competition effect for the binding site, as the presence of Aβ42 in the assay decreased the inhibitory effect of S100B on K18 aggregation (Supplementary Fig. 17). However, further experiments will be required to fully investigate this complex connection.

### S100B interacts with tau fibrillar species inhibiting secondary processes

**Fig. 5:**
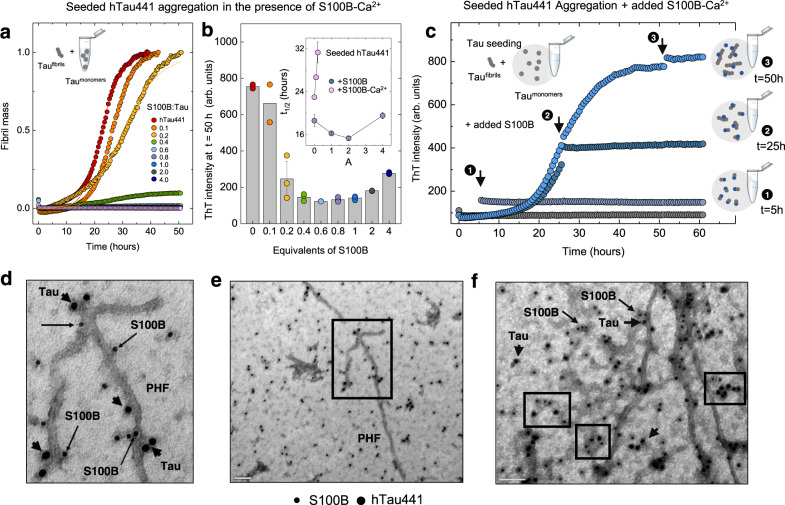
S100B-Ca^2+^ inhibits tau surface-catalyzed secondary nucleation and colocalizes with tau fibrils and oligomers. **a** Seeded fibril formation of 10 µM hTau441 (red) in 50 mM Tris pH 7.4, in the presence of 0.005% of preformed fibrils and 1.1 mM CaCl_2_ at 37 °C under agitation, in the presence of 0.1 (orange), 0.2 (yellow), 0.4 (green), 0.6 (light blue), 0.8 (purple), 1.0 (blue), 2.0 (gray), and 4.0 (dark blue) molar equivalents of S100B. **b** ThT intensity in arbitrary units (arb. units) of the end-time point (same color code) and half-time reaction (inset) of the hTau441-seeded aggregation in the presence (light pink) or the absence (blue) of Ca^2+^, for different S100B molar equivalents. **c** hTau441-seeded aggregation in the same conditions with addition of equimolar S100B at 0 (gray), 5 (light blue, 1), 25 (dark blue, 2), and 50 (blue, 3) hours after aggregation start. Data are presented as mean values ± SD from *n* = 3 independent experiments except for samples with 0.0 and 0.1 equivalents of S100B for which *n* = 2. **d**–**f** TEM images of tau aggregates grown in the presence of S100B-Ca^2+^ and immunogold labeled against S100B (10 nm, arrow) and tau (15 nm, arrowhead). **d** Zoom from the black box in **e**. S100B bound to tau small aggregates and oligomers (boxes in **f**). Scale bar 100 nm (**e** and **f**). Independent experiments of immunogold labeling were performed three times.

We then investigated the effect of S100B using a seeded tau-aggregation assay, to characterize the influence of this chaperone on secondary processes. In this approach, a small amount of preformed tau fibrils is added at the start of the reaction of monomeric hTau441 in the presence of heparin, resulting in faster aggregation and in highly homogeneous kinetic traces that recapitulate the nucleation-dependent polymerization of tau^47^. In agreement, we noted that seeding with 0.005% hTau441 fibrils decreases the reaction half-time ~15 h, from *t*_1/2_ = 23.0 ± 0.2 h versus *t*_1/2_ = 37.9 ± 1.3 h obtained in the unseeded reaction. As observed for the unseeded assays, apo S100B has no effect over seeded aggregation of hTau441. Again, Ca^2+^ is required for the inhibitory action of S100B, and the potency of S100B-Ca^2+^ inhibition is highly increased in seeded assays (Fig. 5a).

Indeed, aggregation of hTau441 is completely abolished at a molar ratio hTau441:S100B > 0.6 (Fig. 5b), which was not the case in the unseeded assay, suggesting that S100B inhibits also secondary reactions that are implicated in seeded hTau441 aggregation. To probe this, we carried out an experiment in which S100B-Ca^2+^ was added at different times along the aggregation reaction of hTau441 (Fig. 5c). When S100B-Ca^2+^ is added in the lag phase (*t* = 5 h), when monomeric tau predominates, the aggregation is abolished because of the interaction between the two proteins, which inhibits primary nucleation. At the reaction half-time (*t* = 25 h), there is already a substantial amount of tau oligomers derived from monomers in fibrils-catalyzed reactions, which constitute nuclei for the fibrillation reaction. When S100B-Ca^2+^ is added at this point, the reaction halts and the ThT intensity does not decrease, suggesting that S100B is also able to interact with tau oligomers and fibrils, and that it does not cause fibril disassembly. This is confirmed when S100B-Ca^2+^ is added at the plateau stage (*t* = 50 h), when formed fibrils predominate, as there is no further change in the fibril mass. To confirm the interaction between S100B and tau fibrillar species and oligomers, we resorted to transmission electron microscopy (TEM) imaging with nanogold-conjugated anti-S100B and anti-tau antibodies. For this, we analyzed hTau441 fibrillar materials formed at the plateau stage upon incubation in the presence of S100B-Ca^2+^ (Fig. 5d–f). In agreement with the mechanistic observations, we observed that S100B (labeled with 10-nm gold particles) binds abundantly along the surface of tau fibrils (labeled with 15-nm particles) as well as to smaller tau aggregates and oligomeric materials (Fig. 5d–f). To further strengthen this conclusion, we carried out a detailed analysis of the preferred binding regions of S100B in a set of tau fibrils to determine if binding occurs preferentially at the extremities of fibrils (which would suggest a preferred inhibitory action over fibril elongation) or indiscriminately along the fibrils, which was found to be the case (Supplementary Fig. 18).

### S100B inhibits seeding potency of tau oligomers

**Fig. 6:**
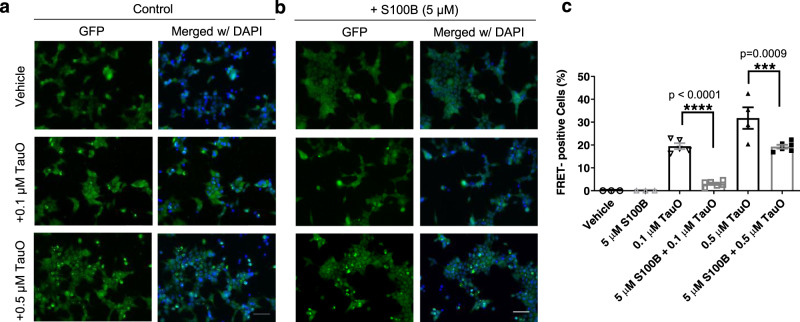
S100B inhibits toxic seeding mediated by tau oligomers. **a**, **b** Representative immunofluorescence images of tau RD P301S FRET biosensor cells exposed to tau oligomers (0.1 µM and 0.5 µM) alone (**a**) or to tau oligomers preincubated with S100B (5 µM) (**b**) for 24 h in the presence of lipofectamine. In all, 5 µM S100B data are shown as representative data of the effect of S100B alone in tau biosensor cells, as even higher concentrations did not cause seeding. Cell nuclei stained with DAPI (blue). Objective used: 40X; scale bar 50 μm. **c** Statistical analysis with data presented as mean and standard error of mean from *n* ≥ 3 independent experimental replicates using one-way analysis of variance (ANOVA) with Tukey’s multiple-comparison test. Specifically, for assays with vehicle and 5 µM S100B *n* = 3, for assays with 0.1 µM TauO *n* = 5, for assays with 0.5 µM TauO *n* = 4, and for assays with 5 µM S100B + 0.1 µM TauO and 5 µM S100B + 0.5 µM TauO *n* = 6. Statistical significance at *p* < 0.05 was considered, ****p < 0.0001 and ***p = 0.0009.

To investigate the role of S100B and tau interaction influencing the seeding capacity of tau oligomers (TauO), we utilized a well-established cell model, tau-RD P301S-CFP/YFP biosensor cells^48,49^. This cell model employs a reporter system in which exogenously added human and mice brain lysates were shown to seed for intracellular tau-inclusion formation. Disease-relevant tau oligomers purified from human AD, DLB, PSP brain tissues, and TBI mice brain tissues as well as recombinant tau oligomers cross-seeded with α-synuclein oligomers, were shown to form tau inclusions in the tau biosensor cells^50,51^. In this study, we exogenously added tau oligomers (TauO, 0.1 or 0.5 µM), S100B (30, 10, 5, 1, and 0.5 μM), and complexes of S100B and TauO prepared at different molar ratios to the biosensor cells (Fig. 6 and Supplementary Fig. 19). These conditions are comparable to those in the AD brain in which S100B is abundantly secreted by astrocytes nearby tau-positive inclusions, reaching high extracellular concentrations^14,52^. As expected, cells treated with vehicle (PBS) or S100B, irrespective of the concentration tested, did not show any tau-aggregate formation (Fig. 6a). The seeding activity of TauO forming tau inclusions was detected by immunocytochemistry using GFP immunofluorescence as bright-green signal measured as FRET-positive cells. Remarkably, S100B and TauO complexes showed differential seeding activity, and the seeding capacity of TauO at both 0.1 µM and 0.5 µM was significantly reduced by its complex with S100B at 5 µM (Fig. 6b, c).

We next tested the effect of different S100B concentrations (0.5, 1, 5, 10, and 30 μM) with 0.5 µM TauO, and observed a decreasing trend in TauO seeding activity, which was significant at the higher tested concentrations (Supplementary Fig. 19). Taken together, our data suggest that the interaction of S100B with tau oligomers inhibits the seeding potency of tau oligomers, thus indicating a regulatory action of S100B on tau oligomers.

## Discussion

Chaperone proteins that act over protein aggregation play critical roles in neurodegeneration, and the activities of core classical chaperones, such as Hsp/DnaJ in the protection against neurotoxic protein deposits, are firmly established^36,53^. Also, Hsp70 was found to have disaggregase activity against tau mature fibrils, reconverting them into monomers^54^. However, chaperone networks include other less conventional chaperones, which are relevant in AD, such as Brichos and clusterin^55–57^. Several studies have also implicated S100 proteins, which have so far been mostly associated with inflammation, in the regulation of neurodegeneration-related protein-aggregation pathways^58,59^. Such findings have prompted us to investigate similar processes in AD, and recent advances include discoveries, which uncovered unique neuroprotective functions that imply S100B as a holdase-type chaperone^21^. Specifically, we established that, under physiological conditions mimicking early disease states, S100B acts as a molecular chaperone, delaying Aβ42 aggregation and mitigating its neurotoxicity^21^. Due to its well-established metal-binding properties, this highly abundant protein also acts as a dual-mode protein–metal chaperone^60^ by combining chelation of zinc and copper, both elevated in AD, with improved suppression of Aβ42 aggregation^18,60,61^. In agreement with its involvement in protective chaperone-like functions, notably at early pre-inflammation disease stages^16^, S100B and other S100 proteins have elevated expression levels and overlapping spatial distribution with protein deposits in AD and in animal models^62^. These observations, along with the fact that S100B is elevated in the brains of mouse models of tauopathies^63^ and the evidence that it is a cellular binding partner of tau^64^, prompted us to investigate its effects over tau.

Initial findings in the early 1980s, based on protein chromatography and cross-linking studies using brain-purified extracts, provided the first indication for a Ca^2+^-dependent inhibitory action of S100B on MT assembly, leading to the hypothesis that a MT-binding protein such as tau might bind to S100B^25^. Here, we have used BiFC analysis, superresolution imaging, and immunocytochemistry to confirm that S100B interacts with tau in MT, and that this interaction is not impaired upon nocodazole-induced MT destabilization. Rather, the S100B:tau interaction persists when tau is released from MT, as shown by its cytosolic accumulation, with formation of puncta-like inclusions. Given that tau is an intrinsically disordered protein with a high propensity to aggregate, these observations suggest that binding of S100B to MT-released tau may afford some protection toward aggregation under pathophysiological conditions. Although S100B is mainly produced by astrocytes^65^, it is also expressed in neurons^66^. Extracellular S100B that accumulates near tau-positive deposits, is also taken up by neurons through endocytosis^18,64,67^.

Here, NMR analysis uncovered the structural details of the interaction between S100B and tau, as well as the mechanism of its biological activation through Ca^2+^ binding. We observed that, in the absence of Ca^2+^, either the S100B:tau interaction does not take place, or occurs only marginally, similarly to what has been reported for several interactions between S100B and other binding partners^68,69^, as well as for its binding and inhibition of Aβ42 aggregation^21^. Indeed, S100B undergoes Ca^2+^-dependent conformational changes that emerge when the two EF-hand domains are occupied. While Ca^2+^ binding to the N-terminal EF hand (EF1) results in minor structural changes^34,70–72^, binding of Ca^2+^ to the C-terminal EF hand (EF2) causes a major conformational change involving helix III (which undergoes a ~90° reorientation) and part of helix IV, opening a hydrophobic protein-interaction site proximate to the dimer interfacial cleft, which is important to target protein binding and recognition^34,69–71^. This agrees with our observation that Ca^2+^ is required for the interaction of S100B with tau, notably with the proline‐rich region at the N-terminal projection domain and the MTBR. Interestingly, these regions have been also identified in regulatory interactions between tau and conventional chaperones such as Hsp90 and DnaJ proteins^36^. Similarly to what we observe for S100B–tau interactions, the association of such classical anti-aggregation chaperones and tau also spans multiple regions along the tau sequence^36^, evidencing the formation of dynamic interactions. This derives from the realization that, as holdase-type chaperones are unable to accomplish full-surface complementarity to all of their clients, they operate by establishing multiple transient local interactions with locally frustrated segments of the client^73^. Therefore, our structural observations provide further support for a holdase-type activity of S100B. Reciprocal NMR analysis of the binding of tau to S100B reveals that preferential interactions occur at the interfacial cleft formed at the dimer interface, involving helix IV from each monomer. It is noteworthy that so far, like in this case, all complexes involving S100B-Ca^2+^ utilize the interfacial helix IV for interactions with target proteins^35,71^, thus reinforcing our observations.

Kinetic analysis of the effect of S100B-Ca^2+^ on hTau441 aggregation indeed reveals a strong inhibitory effect, which is even more notorious when the K18 fragment is used as a model. This peptide harbors the R1–R4 repeat regions from the MTBR and includes the PHF6*/PHF6 hexapeptides with high β-sheet-forming propensity that form the core of tau filaments. The multiple transient interactions between S100B and some segments of the tau/K18 client are characteristic of chaperone–client interactions and involve a permanent rearrangement of the client conformation on the chaperone^73^, completely abolishing tau aggregation at low substoichiometric conditions. Mechanistically, tau aggregation follows a nucleation–conversion–polymerization model^45^ and we observe that, while S100B-Ca^2+^ does not affect the dominant mechanism of tau aggregation, it predominantly affects secondary nucleation rather than fibril elongation or primary nucleation, as corroborated in seeded-aggregation experiments. In agreement with a predominant effect on secondary nucleation, immunogold-labeling TEM experiments show extensive colocalization of S100B along tau fibrils, as well as interactions with tau oligomers. We did not observe preferential binding of S100B at the fibril ends, in agreement with the fact that S100B has decreased effect on fibril elongation.

In this context, the inhibitory action of S100B on tau phosphorylation observed in earlier assays using brain proteins and extracts^26^ can now be rationalized at a detailed molecular and structural level. Indeed, the preferential binding of S100B to the PRR/F5 fragment shields several Thr/Ser residues (including Thr231 in R0 peptide) that would otherwise be targets to phosphorylation by kinases. This constitutes an additional protective mechanism through which the S100B chaperone is not only a potent inhibitor of tau aggregation but a means to prevent tau phosphorylation, whose role in tau release and aggregation is still under debate^74^. A similar mechanism is observed for the interaction of S100B with other protein targets such as p53, whose interaction with S100B overlaps with regulatory phosphorylation sites^75^. Considering that S100B-positive astrocytes are found clustered around and within tau-positive plaques^52^, and that extracellular S100B is present at high levels in AD, this study also suggests a role of S100B in the contention of tau seeding. Using a well-established tau biosensor cell model^49^, we demonstrated that S100B prevents tau-inclusion formation induced by tau oligomers in vitro. This is a key observation regarding the implications of S100B:tau interaction in AD, as it ties such interaction with a neuroprotective effect. Indeed, synaptic tau seeding has been suggested to precede tau pathology in AD, as extracellular soluble tau aggregates are able to recruit and misfold monomeric tau^76^. Extracellular S100B thus may play a protective role by engaging directly proteotoxic oligomeric tau forms.

**Fig. 7:**
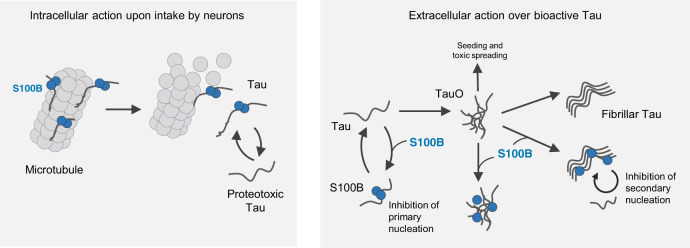
Proposed model for the intra- and extracellular action of the S100B chaperone on tau. The cartoon summarizes regulatory intra- (left) and extracellular (right) interactions established between S100B and tau. Inside cells, S100B operates as a microtubule-regulating factor, likely acting as a protective holdase that mitigates proteotoxic tau, upon its release following microtubule destabilization. Extracellular S100B is increased in the AD brain from early disease stages and is abundantly secreted by astrocytes nearby tau-positive inclusions. In this context, S100B performs as a chaperone inhibiting aggregation through interactions established with released proteotoxic tau, thus contributing to decrease seeding reactions and pathological cell-to-cell spreading.

In conclusion, our findings show that S100B has intra- and extracellular activity as a chaperone counteracting tau aggregation and seeding. Such functions are likely relevant in the context of early inflammatory responses in tauopathies and illustrate how this multifunctional alarmin potentially exerts protective functions against protein aggregation from early disease stages (Fig. 7).

## Methods

### Plasmids

BiFC constructs carrying the 1–158 and 159–238 amino acid fragments of the Venus fluorescent protein were employed to visualize the interaction between tau and S100B in living cells. Tau–Venus BiFC constructs (N-terminal VN tau and C-terminal tau-VC) (available through the Addgene repository, plasmids #87368 and #87369). In the VN-S100B BiFC construct, S100B was fused to the N-terminal fragment of Venus (S100B-VN). Alternatively, S100B was fused to the N terminus of the cerulean fluorescent reporter (full length). Both S100B constructs were synthesized by GeneArt services (Thermo Fisher Scientific, Waltham, MA, USA), and will be deposited in Addgene. The mCherry–Tubulin construct was obtained from Addgene (reference 55148^77^).

### Cell culture, transfection, and treatments

HeLa human cervix adenocarcinoma cells and SH-SY5Y cells were acquired from ATCC (references CRM-CCL-2 and CRL-2266, respectively), and U-251 MG human glioblastoma cells were purchased from ECACC (reference 08061901). All cells were maintained under controlled conditions (37 °C and 5% CO_2_) in DMEM medium (HeLa/U251) or DMEM/F12 (1:1, SH-SY5Y) (Thermo Fisher Scientific, Waltham, MA, USA) supplemented with 10% v/v fetal bovine serum and 1% w/v of a penicillin/streptomycin commercial antibiotic mixture (Gibco, Invitrogen). Cell lines tested negative to mycoplasma contamination using the MycoSpy kit (Biontex Laboratories GmbH, München, Germany). Cells were seeded at a density of 50–60,000 cells/cm^2^ on glass-bottom 35-mm dishes (20-mm observation area, IBIDI, GmBH, Gräfelfing, Germany) for live-cell imaging, and over circular coverslips (Cat. AGL46R13-1, Agar Scientific, Stansted, UK) inside 24-well plates (Cat. 3524, Corning, NY, USA) for immunocytochemistry experiments. Plasmid transfections were carried out 24 h after seeding using JetPrime reagent (Polyplus transfection, Illkirch, France), following the manufacturer’s instructions (1 µg of DNA:4 µl of transfection reagent, using always 1 µg of each construct). HeLa cells were treated with 10 µM nocodazole (Target Mol, Wellesley Hills, MA, USA) diluted in DMSO (100% v/v), 16 h before imaging. MT was stained in living cells using Tubulin Tracker Deep Red following the manufacturer’s instructions (Thermo Fisher Scientific, Waltham, MA, USA). SH-SY5Y cells were transfected with the mCherry–Tubulin construct and differentiated by incubation with DMEM/F12 containing 1% FBS, 10 µM retinoic acid (Cat. R2625, Sigma-Aldrich, St. Louis, MO, USA), and 25 ng/mL brain-derived neurotrophic factor (BDNF, Cat. B3795, Sigma-Aldrich, St. Louis, MO, USA) for five days. Differentiated SH-SY5Y cells were then treated with S100B (30 µM) for 24 h. U-251 MG cells were only submitted to transfection with the corresponding constructs (tau and mCherry–tubulin) 24 h before fixation.

### Co-immunofluorescence

For immunofluorescence, U-251 MG or differentiated SH-SY5Y cells were first fixed with 4% paraformaldehyde (Cat. AP.EM-15710, Electron Microscopy Sciences, PA, USA), permeabilized with 0.1% Triton X-100 (Cat. X100RS, Sigma-Aldrich, St. Louis, MO, USA) and blocked with blocking buffer (2% bovine serum albumin) (Cat. A7030, Sigma-Aldrich, St. Louis, MO, USA) and 5% goat serum (Cat. X0907, Agilent Technologies, Santa Clara, CA, USA). Anti-tau (Cat. sc-58860, Santa Cruz Biotechnology, Dallas, TX, USA) and anti-S100B (Cat. ab-52642, Abcam, Cambridge, UK) antibodies were added at a dilution of 1/100 on blocking buffer and incubated overnight at 4 °C. After washing with phosphate buffer saline (PBS), anti-mouse IgG Alexa Fluor 647-conjugated (Cat. A-21235, Thermo Fisher Scientific, Waltham, MA, USA) and anti-rabbit IgG Alexa Fluor 488-conjugated (Cat. O-6381, Thermo Fisher Scientific, Waltham, MA, USA) secondary antibodies were added at a 1/500 dilution in blocking buffer and incubated for 1 h in a dark humid chamber. For nuclei visualization, cells were counterstained with DAPI (Cat. D9542, Sigma-Aldrich, St. Louis, MO, USA) at 0.5 µM for 10 min. Coverslips were then mounted in slides with mounting medium. Coverslips were sealed and stored at 4 °C until acquisition of fluorescence microscopy images.

### Fluorescence microscopy and image analysis

Live-cell and immunofluorescence images were acquired using either a TCS SP8 (Leica Microsystems, Germany) widefield fluorescent microscope equipped with a Orcha-Flash4.0 Monochrome CMOS camera (Hamamatsu, Japan) or a TCS SPE widefield fluorescent microscope with a CCD camera. Image acquisition was done using the Leica X Core software, and analysis was done using the ImageJ free software (https://imagej.nih.gov/ij/). For time-lapse microscopy, images were acquired every 40 s with a 63x oil-immersion objective, under temperature, CO_2_, and humidity control. For SRRF analysis, images were acquired minimizing the time interval to achieve three shoots per second, until a total of 100 images. NanoJ-SRRF plugin of ImageJ was used to process images^30^, and CellProfiler v4.2.1 was used for the quantification of the puncta^78^ and BoxPlotR online plotting tool (http://shiny.chemgrid.org/boxplotr/) for the representation of violin plots. Images from live or fixed cells were analyzed for colocalization between S100B, tau, DAPI staining, and/or tubulin by means of EZColocalization^79^ or Colocalization finder ImageJ plugins. Briefly, background was subtracted from the S100B, tau, Tubulin, and DAPI channel with rolling-ball radius of 25 pixels. In the S100B channel, the threshold of images was adjusted manually to define automatic regions of interest (ROIs). Colocalization between pairs of channels (tau vs S100B, tau vs DAPI, and tau vs tubulin) was analyzed in the designed ROIs in 224 cells from three independent experiments. The plugins calculated the colocalization coefficient linear Threshold Overlap Score (TOS) and Pearson’s correlation coefficient (PCC), which had a Costes test value of 1. Colocalization values of tau vs S100B were then compared with the colocalization values of tau vs DAPI (negative control, as tau generally has little nuclear localization) and tau vs tubulin (positive control, as tau is a microtubule-binding protein).

### Immunoblotting

Proteins were extracted 24 h after transfection and 16 h after treatment with 10 µM nocodazole. Cells were washed with PBS (2x), lysed in 1% NP-40, 150 mM NaCl, 50 mM Tris, pH 7.4, and a protease-inhibitor cocktail (Roche diagnostics, Basel, Switzerland), and collected by scraping. After 10 min on ice, lysates were sonicated for 10 s at 5 mA, and centrifuged at 10,000 x for 10 min at 4 °C. Protein was quantified using a BCA Protein Assay Reagent Kit (Thermo Fisher Scientific, Inc, Rockford, IL, USA). Equal amounts of protein (~60 µg) were boiled during 5 min in standard loading buffer containing 200 mM Tris-HCl, pH 6.8, 8% SDS, 40% glycerol, 6.3% β-mercaptoethanol, and 0.4% of bromophenol blue. Proteins were then transferred to a nitrocellulose membrane. Ponceau S staining was used to confirm transfer efficiency. Membranes were blocked with 5% nonfat dry milk dissolved in 150 mM NaCl, 50 mM Tris pH 7.4, and 0.5% Tween-20 (TBS-T) for 1 h at room temperature (RT). Membranes were incubated with primary antibody Tau-5 (Cat. sc-58860, dil. 1:200, Santa Cruz Biotechnology, Dallas, TX, USA) and anti-GAPDH antibody (0411) (Cat. sc-47724, dil. 1:30000, Santa Cruz Biotechnology, Dallas, TX, USA). Membranes were cleansed with TBS-T and incubated with a goat anti-Mouse IgG (H+L) Secondary Antibody, HRP (Cat. A16066, dil. 1:10000; ThermoFisher, Waltham, MA, USA) for 1 h at RT. Immunoblots were developed with enhanced chemiluminescence reagents (Millipore, Burlington, MA, EUA) and exposed to X-ray films. Full-scan image of the blot is available at the Source Data file.

### Proteins

hTau441, K18 (tau244–372), F5 (tau165–246), and S100B were expressed in *E. coli* (BL21 DE3). Expression of ^15^N-labeled proteins was performed with M9 minimal medium supplemented with ^15^N-ammonium chloride (CORTECNET, France) as the only nitrogen source. Expression of S100B was performed with Luria-Bertani (LB) medium, and purification was performed by taking advantage of hydrophobic core exposure when binding of calcium ions and using a hydrophobic-interaction chromatography followed by gel filtration^58^. Apo S100B was prepared by incubation at 37 °C for 2 h with a 300-fold excess of dithiothreitol (DTT) and 0.5 mM EDTA followed by purification on a Superdex S75 (GE Healthcare). Expression and purification of recombinant Aβ42 was performed through disruption of Aβ42-expressing cells by sonication followed by resolubilization of the peptide from inclusion bodies by treatment of the lysate with 8 M urea. Aβ42 was then purified in a DEAE-cellulose anionic exchange chromatography (Cytiva, Chicago, IL, USA) followed by a separation in a 30-kDa cutoff filter (Amicon, Merck Millipore, MA, USA)^80^. To obtain the Aβ monomeric form, 1 mg of Aβ42 was dissolved in 7 M guanidine hydrochloride and eluted in a Superdex S75 (Cytiva, Chicago, IL, USA) to remove denaturant excess^21^. The expression and purification of recombinant full-length human tau (hTau441) and the tau fragments K18 and F5 (plasmids pET15b-tau, pET15b-K18, and pET15b-F5) was performed as previously described^44^. Briefly, the transformed cells were selected by plating in Luria-Bertani (LB) solid medium. A single colony was used to inoculate LB medium supplemented with the appropriate antibiotics that was incubated overnight at 37 °C. Bacterial cells were then grown in M9 medium and then induced with IPTG. The cells were harvested after 3 h by centrifugation. The cell pellet was resuspended in buffer A (50 mM phosphate buffer, pH 6.2, and 1 mM EDTA (Sigma-Aldrich, St. Louis, MO, USA)), DNAse (PanReac, Applichem, Darmstadt, Germany), and 1 mM phenylmethanesulfonyl fluoride (PMSF, Roth, Karlsruhe, Germany), and freshly supplemented with complete™ EDTA-free protease-inhibitor cocktail 1x (1 tablet, Roche, Basel, Switzerland). The bacterial cells were disrupted using a high-pressure French Press homogenizer at 20,000 psi, followed by centrifugation to remove insoluble materials. The bacterial cell extract was heated for 15 min at 75 °C in a water bath and then centrifuged again to remove precipitated proteins. Cation-exchange chromatography (CEX) was performed in a HiPrep SP Fast-Flow 16/10 column (Cytiva, Chicago, IL, USA). 50 mM phosphate buffer, pH 6.2, with 1 mM EDTA was used as running buffer and the protein of interest was eluted with the same buffer supplemented with 1 M NaCl. For the aggregation assays, a protocol optimized for producing hTau441 monomers was developed, which consisted in urea (7.6 M) solubilization of post-chromatography fractions in the presence of 50 mM DTT, prior to passage on gel filtration while using a Tricorn Superdex S-200 column. The eluted monomeric tau samples were then lyophilized and stored at −20 °C. Tau purity was assessed by mass spectrometry and SDS-PAGE. Protein concentration was determined spectrophotometrically.

### NMR spectroscopy

NMR experiments were recorded at 298 K on Bruker 900-MHz spectrometer equipped with a triple-resonance cryogenic probe (Bruker, Karlsruhe, Germany). 100 µM ^15^N-tau in a volume of 200 µL in 3 mm tubes was enough to obtain the 2D ^1^H^15^N HSQC (heteronuclear single quantum correlation) spectra with 32 scans, with assignments done as per Biological Magnetic Resonance Data Bank (BMRB) Entries 17945 and 50701 and published data^81,82^. To map the interaction between ^15^N-tau and S100B proteins (100 µM:120 µM final concentrations), 2D spectra were recorded of samples in NMR buffer (50 mM Hepes, pH 6.8, with 1 mM DTT, complete™ EDTA-free protease-inhibitor cocktail 1x (Sigma Aldrich, Saint Louis Missouri, USA), and 10% D_2_O) with either 1.25 mM EDTA or 250 µM CaCl_2_. For the S100B mapping (^15^N-S100B), NMR measurements were made in NMR buffer with 1.25 mM CaCl_2_. 1 mM d4-TMSP (3-(trimethylsilyl)) propionate was used as an internal reference for proton chemical shifts (0 ppm). Two-dimensional ^1^H^15^N HSQC spectra were processed using Bruker TopSpin software (version 3.6, Bruker, Karlsruhe, Germany), and analyzed using Sparky software (version 3.6, T. D. Goddard and D. G. Kneller, University of California, San Francisco, CA, USA) by NMRFAM Sparky (Woonghee Lee, Univ. Wisconsin-Madison, USA). S100B assignment was transferred to our experimental conditions from BMRB 27199^21^ by a three-point temperature titration (277 K, 288 K, and 298 K) at 1.25 mM CaCl_2_ concentration (Supplementary Fig. 5). HSQC spectra were used to monitor changes in amide chemical shift perturbations, corresponding to intensity variation or value modification. The combined chemical shift value changes of resonances, ∆δ(^1^H,^15^N), were calculated using the following equation: ∆δ(^1^H,^15^N) = (∆δ^2^_H_ + ∆δ^2^_N_ × 0.2)^1/2^. The NMR data generated in this study have been deposited in the Zenodo database^83^.

### Paramagnetic relaxation enhancement experiments (PRE)

For these experiments, the tau MTBD natural cysteines (Cys292 and 322) were modified. The location of the probes is located within the identified S100B-binding sites (Fig. 2). The 3-(2-iodoacetamido)-2,2,5,5-tetramethyl-1-pyrrolidinyloxy radical (iodoacetamido-PROXYL, Sigma-Aldrich) was dissolved in DMSO (100 mM) and added to the protein sample (70 µM) at 1 mM final concentration in 50 mM ammonium bicarbonate and 1 mM THP. The excess of the spin label was quenched by adding 5 mM DTT and removed by buffer exchange against 50 mM ammonium bicarbonate (PD10, G25 resin, Cytiva). After lyophilizing, the protein was dissolved in NMR buffer with 1.25 mM CaCl_2_ and mixed for NMR experiments with S100B protein at final concentrations of 180 μM spin-labeled MTBD and 120 μM ^15^N-S100B. The interaction between the nuclei and the unpaired electron on the paramagnetic probe causes an increase in the nuclear relaxation rates, which is reflected in a decrease of intensity of the resonances of the neighboring residues within a radius of 35 Å. PRE effects on ^15^N-S100B protein were quantified by intensity ratios of cross-peaks in the presence of labeled MTBD in diamagnetic and paramagnetic (spin-tagged) states. Diamagnetic state referred to the sample treated by 2.5 mM vitamin C, as reducer of the nitroxide label.

### Binding analysis of Tau peptides

Twelve-residue-long Tau peptides were purchased from Genecust (France) and were capped at their N terminus by acetylation and C terminus by amidation. The sequences are the following R0 peptide: R E P K K V A V V R T P, R2 peptide P G G G K V Q I I N K K, R3 peptide P G G G S V Q I V Y K P, and R5 peptide: K K I E T H K T F R E N. Sequences of the PHF6* in R2 and PHF6 in R3 are underlined. Titration of 100 μM ^15^N-S100B with increasing amount of tau peptides RO, R2, and R3 (from 50 µM to 750 µM) was performed in 50 mM Hepes, pH 6.8, 1.25 mM CaCl_2_ by monitoring the complex formation using the gradual ^1^H and ^15^N chemical shift change Δδ(^1^H,^15 ^N) of the resonances in ^1^H,^15^N HSQC, at 298 K. Dissociation constants were obtained by fitting the chemical shift perturbation data to the following equation: ${\Delta \delta }_{{obs}}=\frac{0.5{\Delta \delta }_{{\max }}}{a\left[a+b+{K}_{d}-\big(\big(a+b+{K}_{d}-{\left({\left(a+b+{K}_{d}\right)}^{2}-\left(4{ab}\right)\right)}^{\frac{1}{2}}\right]}$, where ${\Delta \delta }_{{obs}}$ is the average weight of the chemical shifts in the free and bound states and ${\Delta \delta }_{{\max }}$ is the maximal signal change upon saturation. K_d_ is the dissociation constant, and *a* and *b* are the total peptide and S100B concentrations, respectively. Dissociation constants (K_d_) were averaged, and standard deviations provided, based on chemical shift perturbation analysis of 11 distinct resonances.

### Small-angle X-ray scattering

SAXS measurements were performed on beamline B21 at Diamond Light Source, Didcot, UK. Size-exclusion chromatography (SEC)-coupled SAXS data were recorded using a Superdex 200 (3.2/300) column using 50 mM Tris-HCl, 100 mM NaCl, 2% glycerol, and 2 mM CaCl_2_, pH 7.9, as running buffer. S100B alone, S100B mixed with equimolar K18, or K18 alone, were analyzed. 50 µL of each sample with a concentration of 12–13 mg/ml were injected onto the column. Buffer subtractions and all other subsequent analysis were performed with the program ScÅtter (http://www.bioisis.net). The SAXS curves were fitted with GNOM, and 3D envelopes were reconstructed either with DENSS or with DAMMIF. For analysis with DENSS, the DENSSWeb server (https://denss.ccr.buffalo.edu) was used running 20 individual DENSS reconstructions and subsequent alignment and averaging^84^. For analysis with DAMMIF, 13 independent reconstructions were aligned and averaged using DAMAVER. SUBCOMB and SITUS^85^ were used to align the 3D reconstructions and fit the X-ray structure of S100B dimer (PDB: 2H61) into the 3D envelopes. GNOM, DAMMIF, DAMAVER, and SUBCOMB are part of the ATSAS v3.0.4 software suite^86^. Figures were prepared with ScÅtter and Chimera v1.15^87^. The SAXS data-collection and scattering-derived parameters are provided as Supplementary Table 1.

### Far-UV circular dichroism

CD measurements were performed on a Jasco J-1500 spectropolarimeter (MD, USA) using the Spectra Manager software (v2.13, Jasco, MD, USA). In all, 200-µL samples were measured in far-UV CD cuvettes with a 0.1 cm of optical pathway (Müllheim, Germany) programmed to measure with a wavelength range from 200 to 260 nm, eight accumulations, 0.5 nm of data pitch, 1-nm bandwidth, 100 nm/min of scanspeed, and a programmed temperature of Peltier-controlled thermostated cell support of 20 °C. S100B was used at 5 µM and K18 from 1 µM to 5 µM, in the presence or absence of 1.1 mM CaCl_2_, samples were prepared using 50 mM Tris-HCl, pH 7.4, chelexed buffer. After sample preparation, it was incubated for 1 h at 4 °C before measurement.

### ANS-fluorescence binding

Anilinonaphthalene-8-sulfonic acid (ANS) binds to hydrophobic patches of proteins, enhancing its fluorescence coupled to a blue shift in the emission spectra. ANS was used to probe the binding between S100B-Ca^2+^ and hTau441 and the fragment K18. For that, 10 µM S100B in 50 mM Tris pH 7.4, 1.1 mM CaCl_2_ was titrated with different concentrations (0.5–50 µM) of hTau441 or K18. Samples (350 µL) with 100 µM ANS were prepared and incubated at least 30 min at room temperature before measurement in a quartz cuvette (Hellma, Müllheim, Germany) at 25 °C in a spectrofluorometer (Jasco FP-8200, Tokyo, Japan) with excitation set to 380 nm. The binding fraction was calculated through the normalization of intensity-average emission wavelength^88^ that resulted from fluorescence acquisition. To estimate apparent dissociation constants, data were fitted to the one-site binding model of OriginPro version 2019b (MA, USA).

### hTau441 and K18 aggregation kinetics

hTau441 and K18-aggregation kinetics were performed by recording the thioflavin-T (ThT) fluorescence intensity as a function of time in a plate reader (FLUOstar OPTIMA, BMGLabtech, Ortenberg, Germany) with OPTIMA v2.20 and MARS Data Analysis version 2.10 software and equipped with a 440-nm excitation filter and a 480-nm emission filter. The fluorescence was recorded while using bottom optics in half-area 96-well polyethylene glycol-coated black polystyrene microplates with a clear bottom (Cat. 3881, Corning, NY, USA). The microplates were sealed with transparent foil to avoid evaporation. Concentrations in samples for the aggregation assays were 25 µM hTau441 or 10 µM K18 and 500 µg/mL (~27.8 µM) heparin (Cat. H3149, Sigma, MO, USA) to induce hTau441 aggregation, or 90 µg/mL (~5 µM) for K18 aggregation. For arachidonic acid- (AA, Cat. SML1395, Sigma-Aldrich, St. Louis, MO, USA) induced K18 aggregation, AA was used at a final concentration of 375 µM. Other components, when present were 1 mM DTT (Cat. A1101, Applichem, Darmstadt, Germany), 50 mM NaCl (Cat. MB15901, NZYTech, Lisbon, Portugal), 75 µM ThT (Cat. T3516, Sigma, MO, USA), or 0.5 µM p-FTAA (kind gift from the VIB-SWITCH Lab, KUL, Belgium) and 1.1 mM CaCl_2_ (Cat. 21100, Sigma, MO, USA). In experiments in the presence of S100B, the used concentration ranged from 0.1 µM to 100 µM. S100B concentration is described as molar equivalents in the legend of the figures. Experiments with Aβ42 were carried out at a final concentration of 5 µM.

For the hTau441-seeded aggregation: 10 µM hTau441 was used in the same conditions as mentioned above, except for the addition of 0.005% of preformed sonicated hTau441 fibrils. hTau441 seeds were produced after incubation of 50 µM hTau441 at 37 °C in the same conditions as above for 200 h. The hTau441 fibrils were recovered by centrifugation and were resuspended in the same buffer volume. The fibrils were sonicated with a Hielscher UP200S ultrasonic sonicator 200 Watts (Hielscher Ultrasonics, Teltow, Germany) at 30% for 15 s with 1 s on and 2 s off (as described in^47^). After sonication, this solution was considered as 100% hTau441 seed solution and was flash-frozen and kept at −80 °C until use.

For the K18-seeded aggregation: 10 µM K18 was used in the same conditions as above, but screening in addition, a range of concentrations from 0.005 to 10% of preformed sonicated K18 fibrils. K18 seeds were produced after incubation of 40 µM of K18 at 37 °C in the same conditions as above for more than 150 h. The same conditions were employed for either K18 fibrils or hTau441 seed formation.

A Chelex resin (Bio-Rad, CA, USA) was used to remove contaminant trace metals from all solutions. Samples were prepared while using 50 mM Tris-HCl, pH 7.4. The aggregation kinetics was performed at 37 °C with an orbital shaking at 600 rpm for 300 s before each measurement or in quiescent conditions. Three (or two) independent replicates were performed for each of the tested conditions and are presented in the plots as the average values and standard deviation. Aggregation kinetic data were analyzed using the online platform AmyloFit^89^, which implements the master equations derived from basin-hopping algorithm. This equation describes the evolution of total fibril mass in the presence of primary and secondary nucleation events, and allows microscopic processes and reaction rates to be determined by global fitting of the data^89^. The kinetic curves were fitted using the best-describing model, i.e., the secondary nucleation-dominated model.

### Atomic force microscopy

The topography/morphology of the samples was characterized by AFM with a PicoSPM LE (Molecular Imaging) system and Agilent Technologies PicoView 1.14.4 software (Keysight Technologies, Santa Rosa, CA, USA). The images were obtained in air, at RT, first with HQ:NSC 35/Hard/Al BS (force constants 5.4 N/m, 8.9 N/m, and 16 N/m, tip radius <20 nm) and the selected samples with HiRes-C14/Cr–Au (force constant 5 N/m, tip radius <1 nm) µmasch cantilevers in dynamic mode. Approximately 10 µL of each tau solution was deposited onto freshly cleaved mica (Agar Scientific, Stansted, UK). Before imaging, the samples were allowed to rest for about 20 min, rinsed with water, and then dried in air at RT. For each sample, the present structures were systematically imaged, and the images were analyzed. Images were processed using Gwyddion v2.56.

### TEM and immunogold labeling

For morphological analysis, 5-μl sample aliquots were adsorbed to carbon-coated collodion film supported on 300-mesh copper grids and negatively stained with 1% uranyl acetate. For immunogold labeling, 5-μl sample aliquots were adsorbed to carbon-coated collodion film supported on 300-mesh nickel grids for 5 min. After washing with phosphate-buffered saline (PBS), the grids were blocked with 1% bovine serum albumin (BSA) in PBS for 10 min, and then incubated with anti-S100B monoclonal antibody from rabbit (dil. 1:50, Cat. ab52642, Abcam, Cambridge, UK); for double labeling, grids were sequentially incubated with anti-S100B antibody, followed by anti-tau antibody (Tau-5) monoclonal from mouse (dil. 1:50, Cat. sc-58860, Santa Cruz, Biotechnology, Dallas, TX, USA), diluted in 1% BSA/PBS. Following washing with PBS, grids were then either incubated with anti-rabbit (dil. 1:20, Cat. EM.GAR10, BBI Solutions) or, in case of double labeling, sequentially incubated with anti-rabbit and anti-mouse (dil. 1:20, Cat. EM.GMHL15, BBI Solutions) secondary antibodies conjugated to 10- or 15-nm colloidal gold, respectively. Following washing with PBS, samples were negatively stained with 1% uranyl acetate. The grids were visualized with a JEOL JEM-1400 transmission electron microscope equipped with an Orious Sc1000 digital camera, and exhaustively observed. As a control, we performed the immunogold labeling, incubating only the secondary antibodies, and no colloidal gold was observed. Independent experiments of both morphological analysis and immunogold labeling were performed at least three times, and images presented are representative of the results obtained.

### Seeding assay in Tau RD P301S biosensor cells

The seeding assay of tau oligomers (TauO), S100B and TauO–S100B complex, was carried out in Tau RD P301S CFP/YFP biosensor cells (ATCC, Cat. CRL-3275) as described previously^51^. Briefly, biosensor cells were cultured in DMEM supplemented with 10% FBS, 100 μg/mL penicillin, and 100 μg/mL streptomycin and maintained at 37 °C in a humidified atmosphere equipped with 5% CO_2._ Cells were plated in poly-L-Lysine-coated coverslips in 24-well plates at 1 × 10^5^ cells/well. After 18 h, cells were transduced with seed-transduction complexes prepared by separately incubating TauO (0.1 and 0.5 µM), S100B (10 and 30 µM), and complexes of S100B and TauO and complexes of S100B and TauO (S100B [5 µM]/TauO [0.1 µM]; S100B [5 µM]/TauO [0.5 µM]; S100B [30, 10, 5, 1, and 0.5 µM]/TauO [0.5 µM], separately) with Liposome (Lipofectamine 2000, Cat. 11668-027, Invitrogen, MA, USA) mixed in Opti-MEM (Cat. 31985-070, Gibco, Invitrogen, MA, USA) as previously published^48,51^. The complexes of S100B and TauO were prepared by mixing them at their above-mentioned molar concentrations and incubating at RT for 1 h. Before treating the cells, all proteins, including S100B/TauO complexes, were separately incubated with Liposome at RT for 30 min. Cells were treated with the transduction complexes for 24 h. After treatment, cells were washed three times with PBS and the coverslips were fixed with 4% formaldehyde and mounted with Prolong Gold mounting media for imaging. Cells were imaged with a Keyence BZ-800 Epifluorescence Microscope using Nikon 40X objective. Cells containing bright GFP signal generated by tau inclusions were measured as FRET-positive cells. Statistical analyses were performed using Prism 6.0 (GraphPad Software, Inc., San Diego, CA, USA). Data are presented as mean and standard error of mean (SEM) from at least three replicates using one-way analysis of variance (ANOVA) with Tukey’s multiple-comparison test. Statistical significance at *p* < 0.05 was considered.

### Reporting summary

Further information on research design is available in the Nature Research Reporting Summary linked to this article.

## Supplementary information

Supplementary Information

Description of Additional Supplementary Files

Supplementary Movie 1

Reporting summary

## Data Availability

The NMR data generated in this study have been deposited in the Zenodo database under accession code DOI:10.5281/zenodo.5511948 [https://zenodo.org/record/5511948#.YU4ZwJrMKUl]. All other data generated in this study are provided in the Source Data file. Source data are provided with this paper.

## Source data

Source Data
