# Supplementary material for: Dynamic interactions and Ca2+-binding modulate the holdase-type chaperone activity of S100B preventing tau aggregation and seeding
Source: Nat Commun. 2021 Nov 1;12:6292. doi: 10.1038/s41467-021-26584-2 (PMC8560819; doi:10.1038/s41467-021-26584-2)
Supplement: Supplementary file 1 — Supplementary Information [file 41467_2021_26584_MOESM1_ESM.pdf]

Supplementary information for

Dynamic interactions and Ca^{2+}-binding modulate the holdase-type chaperone activity of S100B preventing tau aggregation and seeding

Moreira et al.

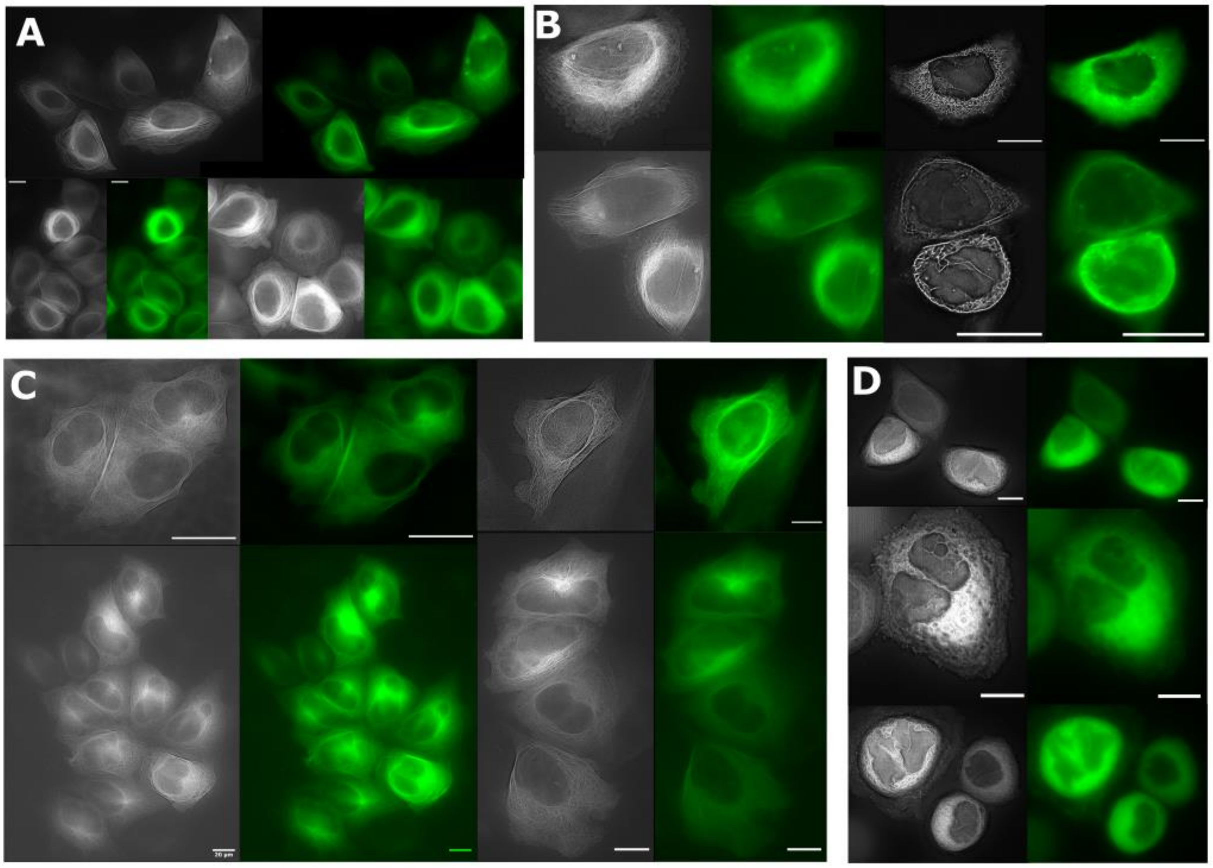

Supplementary Figure 1 - Widefield microscopy and SRRF reconstruction images showing S100B and tau colocalization in living cells.

Representative Widefield microscopy and SRRF reconstruction images of HeLa cells transfected with tau/tau (**A** and **B**) and S100B/tau (**C** and **D**) BiFC constructs. Cells were imaged after 24 hours of transient transfection with no treatment (**A** and **C**) and following washout cleansing with fresh medium after treatment with 10 µM nocodazole for 16 h. Images are representative of n = 4 independent experiments with similar results, and total of 927 cells for tau/tau combinations and 873 for tau/S100B were analysed. Scale bar 10 µm.

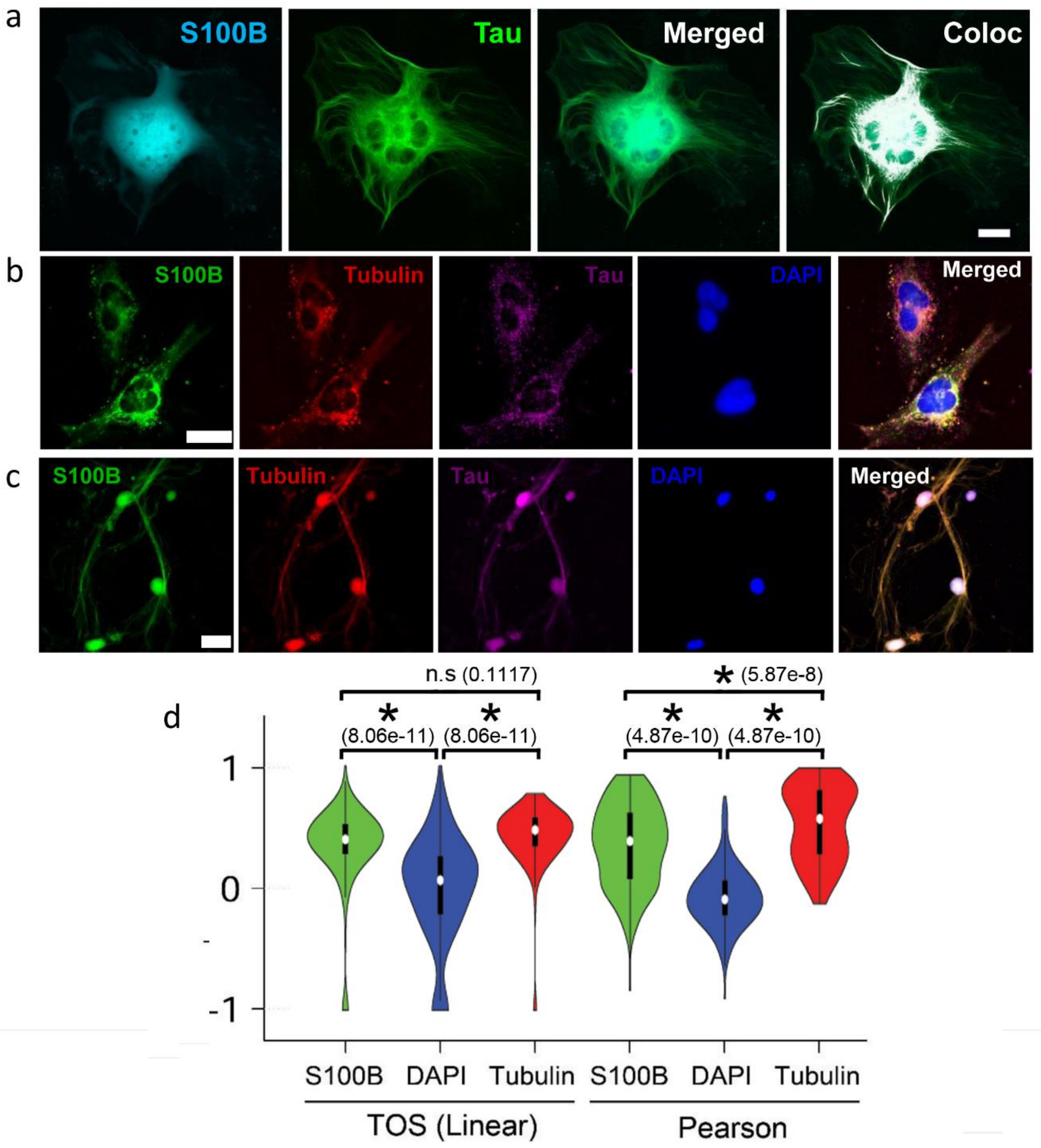

Supplementary Figure 2 - S100B and tau colocalize in living and fixed cells.

a) HeLa cells were co-transfected with the tau/tau BiFC pair (green) and a pcDNA-S100B-Cerulean (blue) construct (1 µg each) for 16 hours to confirm the co-localization of these proteins in living cells. Images were analysed by means of the Colocalization Finder ImageJ plugin (White dots) and showed Pearson´s coefficient R values (PCC) between 0.70 and 0.86. As expected of a dynamic, reversible interaction, co-localization is partial. Scale bar: 20 µm. **b)** U251 human glioblastoma cells were transfected with a single tau construct (non-fluorescent) and an mCherry-Tubulin construct (red) (1 µg each) for 16 hours and fixed. Immunofluorescence was carried out with antibodies against S100B (green) and tau (far-red, purple). Cells were counterstained with DAPI. Images are representative of n = 3 independent

experiments with similar results, and a total of 224 cells were analysed. Scale bar: 20 µm. **c)** SH-SY5Y human neuroblastoma cells were transfected with the mCherry-tubulin construct (red) (1 µM), differentiated for 5 days with retinoic acid (10 µM) and BDNF (25 ng/mL), and incubated for 24 hours with recombinant S100B (30 µM) before fixation. Immunofluorescence was carried out with antibodies against S100B (green) and tau (far-red, purple). Cells were counterstained with DAPI. Scale bar: 20 µm. **d)** Images from SH-SY5Y experiments were analysed by means of the EZColocalization ImageJ plugin (224 cells from 3 independent experiments). Violin plots represent the distribution of the TOS (linear) and Pearson co-localization coefficients of tau versus S100B (green), DAPI staining (blue, negative control) and Tubulin (red, positive control). Average colocalization levels of S100B and tau were positive (Threshold Overlap Score, TOS, 0.34 ± 0.34; PCC, 0.35 ± 0.33; where errors are represented as the standard deviation), significantly higher than between tau and DAPI (TOS, -0.002 ± 0.43; PCC, -0.075 ± 0.24) and closer to the colocalization between tau and microtubules (TOS, 0.42 ± 0.26; PCC, 0.53 ± 0.30). Plots show the median (white dot) and 1st and 3rd quartiles (floating black boxes). Minimum and maximum values of the distribution are shown as the bottom and top of the violin plot. Data were analysed by means of a One-way ANOVA followed by a Bonferroni test *, significant versus the indicated groups, p<0.001. n.s. not significant.

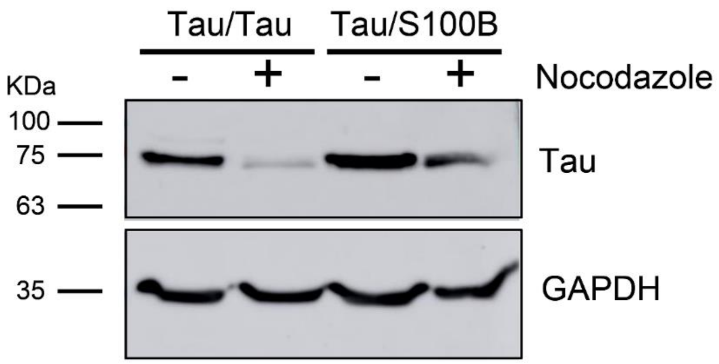

Supplementary Figure 3 - Western blot of tau levels on HeLa cells from BiFC experiments.

Immunoblot analysis of tau expression using an antibody targeting total tau (Tau-5). Levels of expression are similar when cells are transfected with tau/tau and S100B/tau BiFC system (lanes 1 and 3). Treatment with 10 µM of nocodazole for 16 h decreases the quantity of tau on both BiFC systems but remains similar between them (lanes 2 and 4). Images are representative of at least n = 3 independent experiments with similar results.

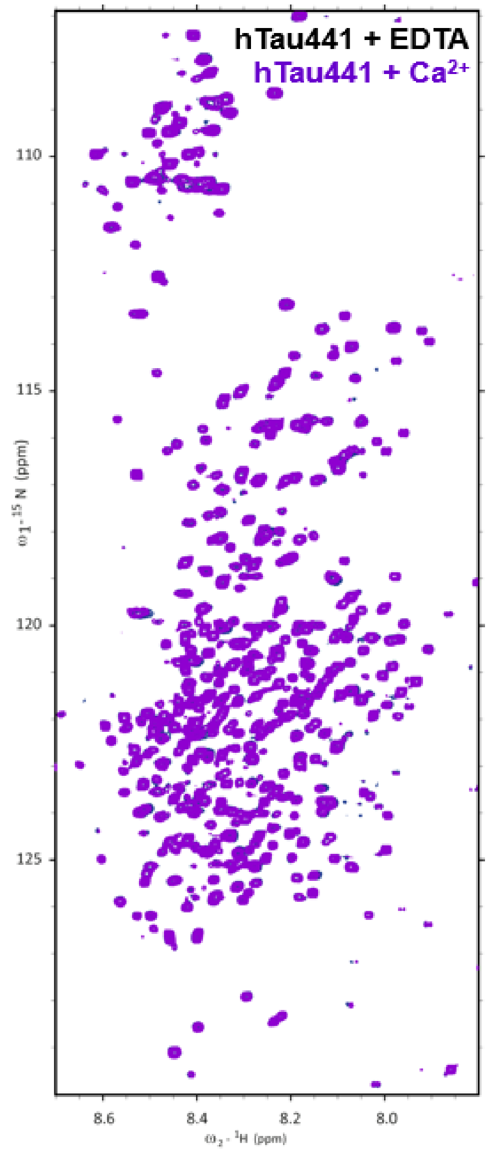

Supplementary Figure 4 - 2D $^{1}H,^{15}N$ HSQC spectrum of full-length tau (hTau441) in the apo state and in the Ca^{2+}-bound state.

2D $^{1}H,^{15}N$ HSQC spectra of 100 µM ^{15}N-hTau441 in 50 mM Hepes buffer pH 6.8 with 1.25 mM EDTA (black spectrum) or with 250 µM $CaCl_2$ (superimposed in purple).

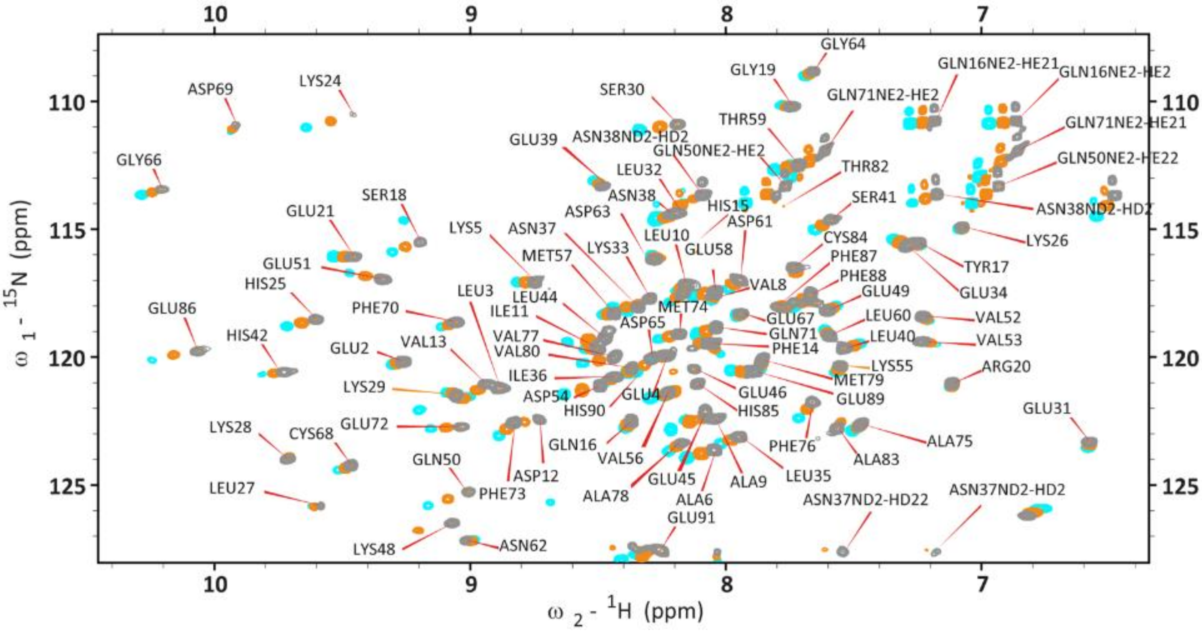

Supplementary Figure 5 - S100B NMR analysis of temperature titration.

Superimposed 2D ^{1}H,^{15}N HSQC spectra of 100 µM ^{15}N-S100B protein with 1.25 mM $CaCl_2$: at 298 K (gray spectrum), at 288 K (orange spectrum) and at 277 K (blue spectrum). Labelling is for the 298 K conditions used in this study.

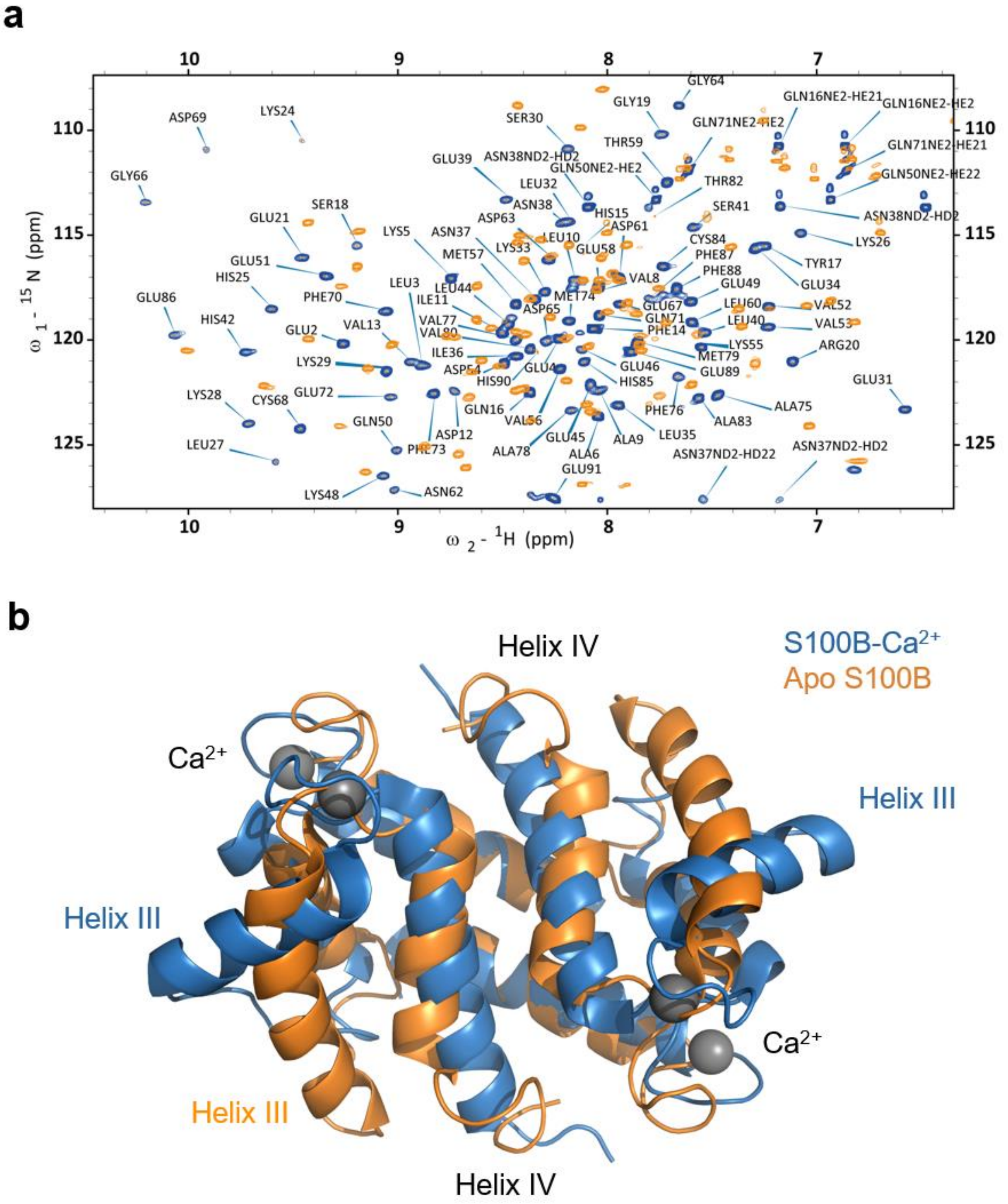

Supplementary Figure 6 - Effect of Calcium on the S100B NMR spectrum.

a) Comparison of the ^{15}N-S100B 2D spectra in the holo-state, obtained in presence of 1.25mM $CaCl_2$ (in blue) and in the apo-state without calcium, obtained in the presence of 2.5 mM EDTA (orange). The modification of the chemical shift values for all the resonances of S100B is indicative of a global conformational rearrangement depending on calcium binding. **b)** cartoon depicting the conformational changes occurring upon Ca^{2+}-binding to human S100B, drawn using Pymol from PDB structures 2PRU (apo) and 2H61 (holo).

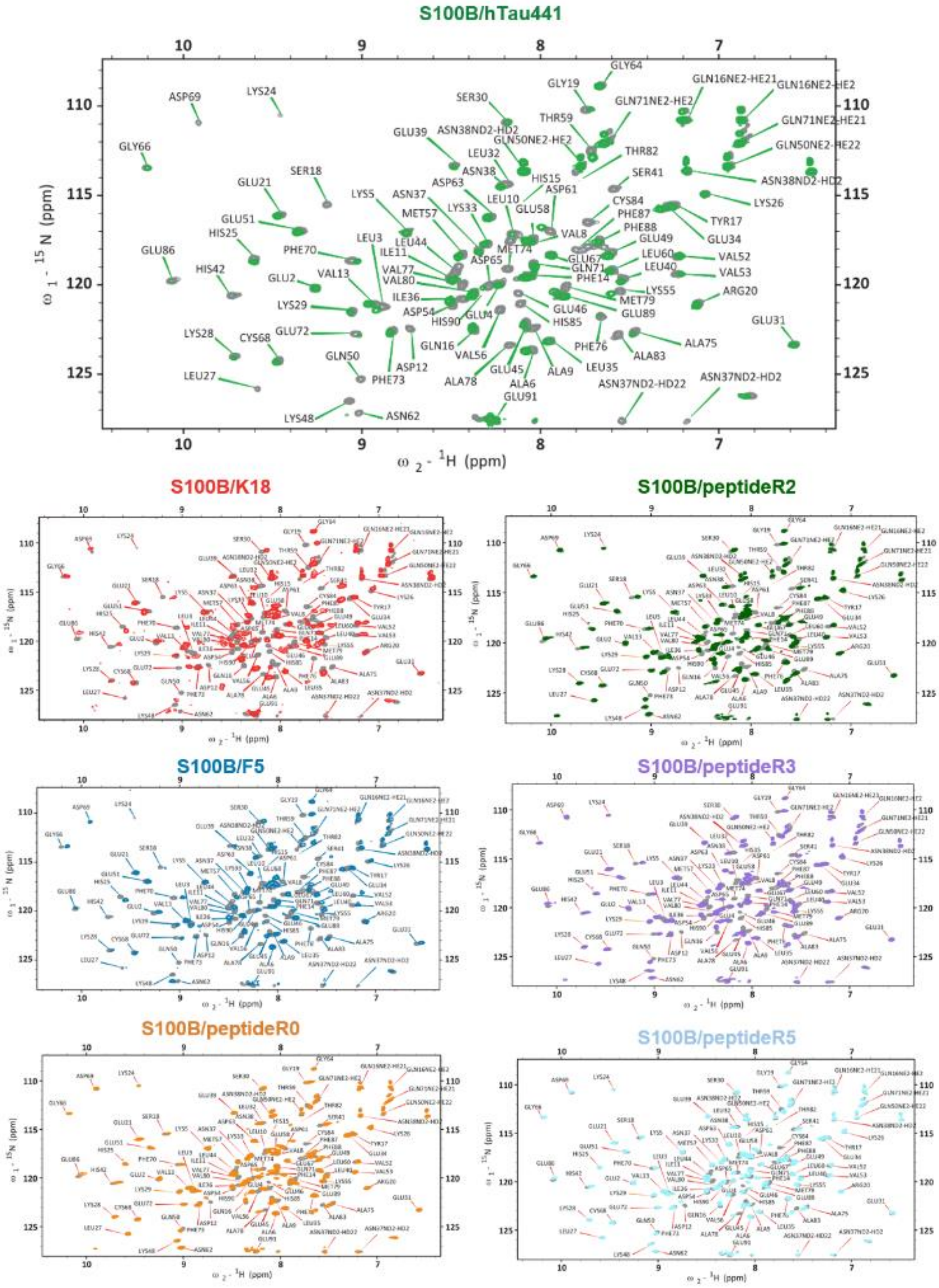

Supplementary Figure 7 - 2D $^{1}H,^{15}N$ HSQC spectra of S100B in the presence of Tau441, fragments F5 and K18 and peptides R0, R2, R3 and R5.

2D overlayed $^{1}H,^{15}N$ HSQC spectra of 100 µM ^{15}N-S100B protein in 50 mM Hepes buffer, pH 6.8 with 1.25 mM $CaCl_2$ (grey spectrum) with: 160 µM hTau441 protein (light green); 100 µM K18 protein fragment (red); 160 µM F5 protein fragment (blue); 250µM of tau peptides R0 (orange), R2 (dark green), R3 (purple) and R5 (cyan).

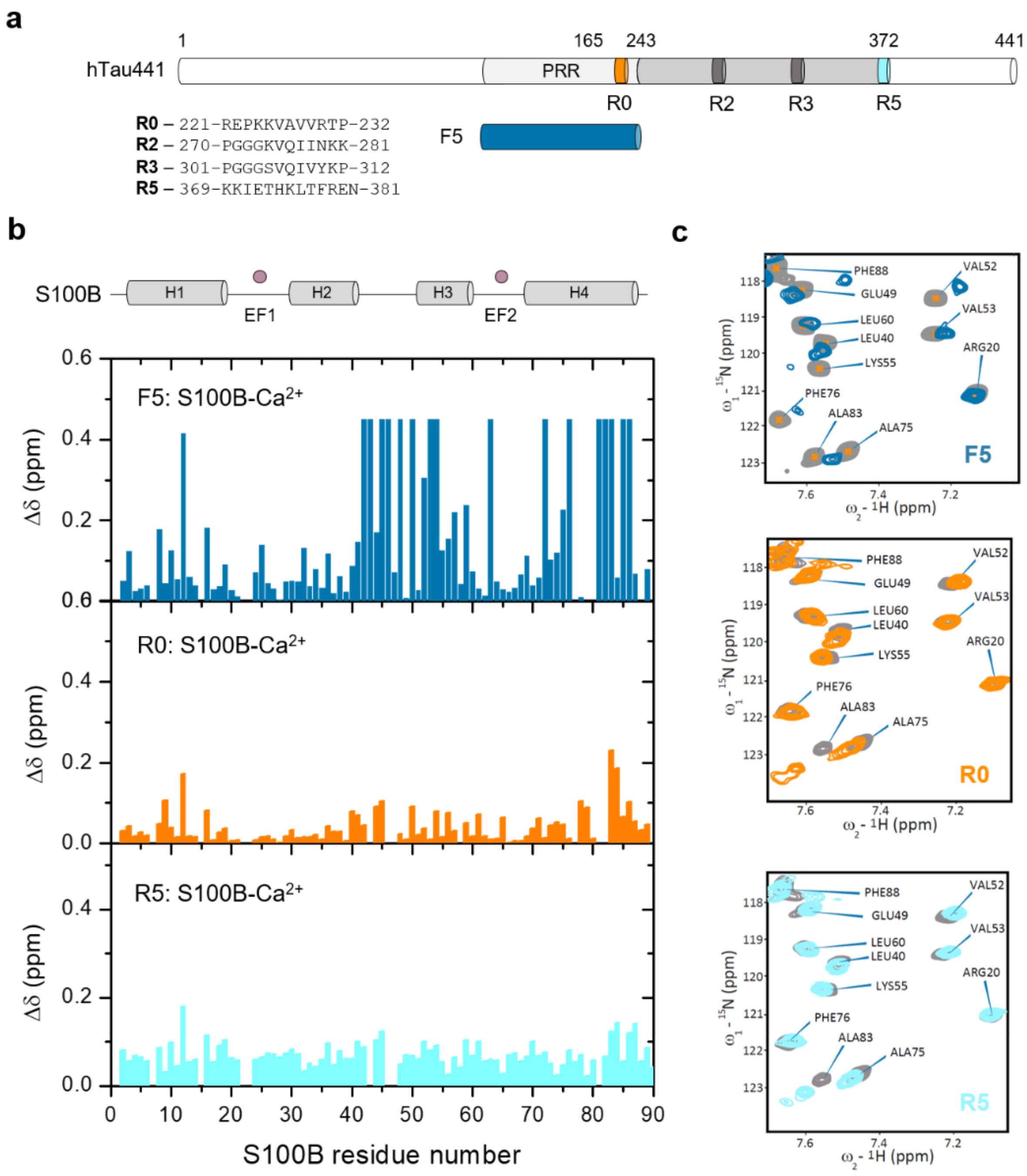

Supplementary Figure 8 - Chemical shift perturbation of S100B residues upon interactions of tau fragments and peptides

a) Schematic representation of full-length tau highlighting the location of the F5 Fragment and of the R0, R2, R3 and R5 peptides. **b)** Chemical shift perturbation of 100 µM ^{15}N-S100B residues in presence of 1.25 mM $CaCl_2$ and 160 µM F5 (containing PRR, blue), 250 µM of the R0 peptide (orange) and 250 µM of the R5 peptide (light blue). **c)** zoom of HSQC spectra evidencing changes upon tau-F5 (blue), peptide R0 (orange) and peptides R5 (cyan) binding to S100B-Ca^{2+} (grey).

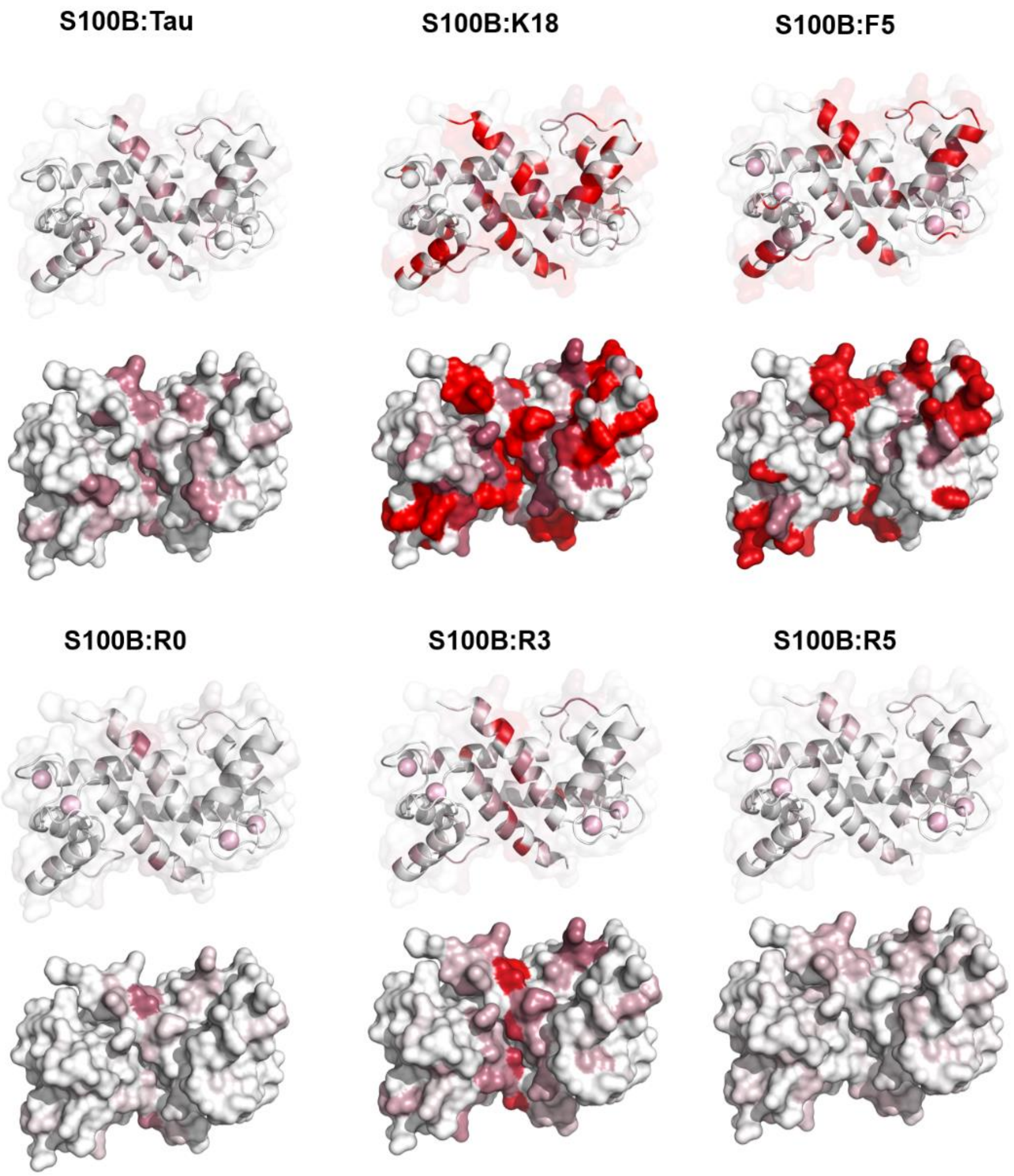

Supplementary Figure 9 - Residue mapping of tau interactions to S100B structure.

Structural mapping on the S100B structure (PDB: 2H61) of the chemical shift perturbations observed upon interactions with hTau441, tau fragments (F5 or K18) and peptides (R0, R3 and R5). S100B residues are color-coded by the variation of the combined ^{1}H, ^{15}N chemical shift values, from red ($\Delta\delta$ (ppm) = 0.5) to white (minimum, $\Delta\delta$ (ppm) = 0.05). Ca^{2+} are represented as pink spheres. Drawn using Pymol.

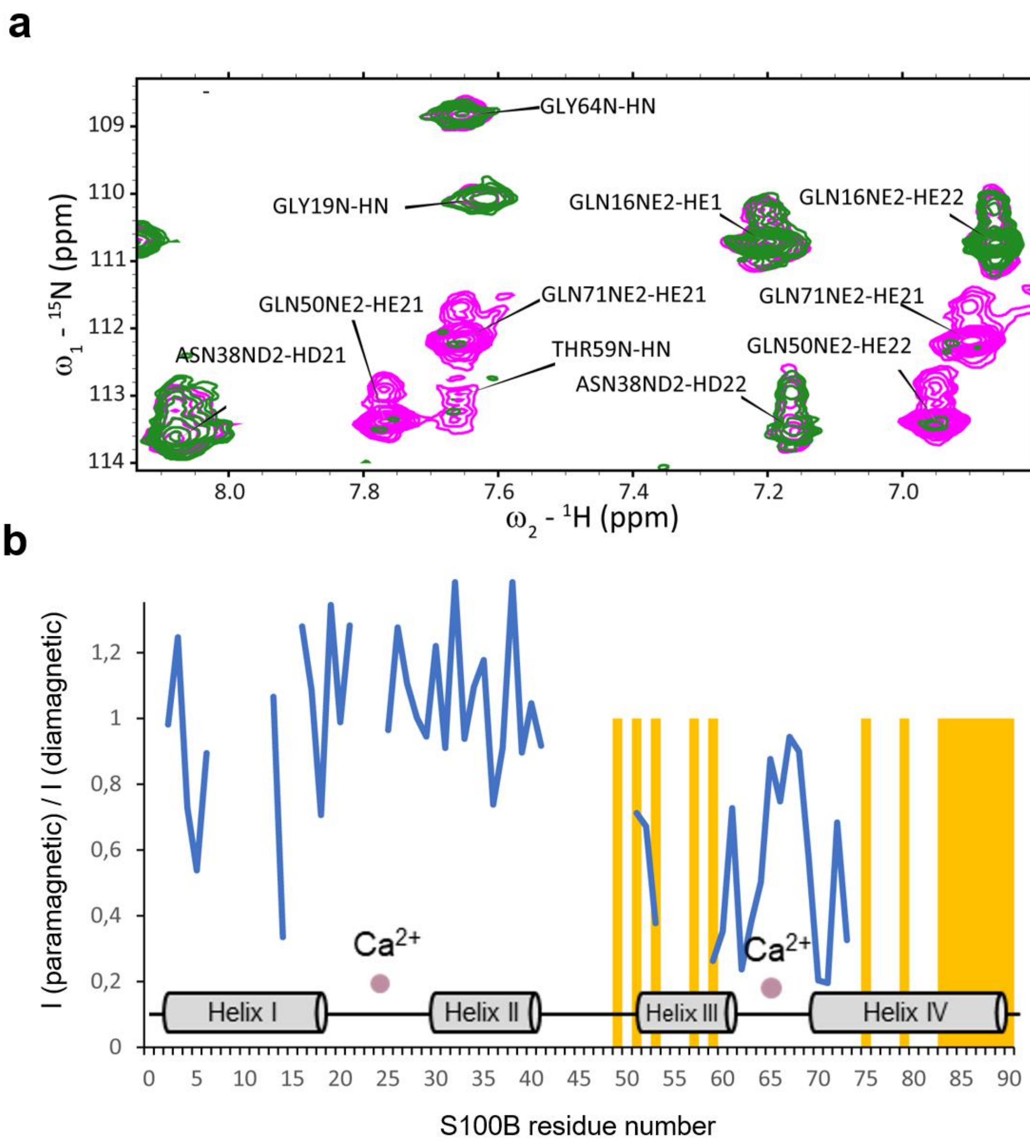

Supplementary Figure 10 - NMR relaxation enhancement experiment

a) Overlay of enlarged regions of ^{15}N-S100B in the presence of nitroxide labelled MTBR (in green, paramagnetic state) and in the presence of reduced nitroxide labelled MTBR (in pink, diamagnetic state). Resonances are broadened when they are located within the radius of action of the paramagnetic probe (about 35 Å) attached to the Cys292 and Cys322 of the MTBR: see resonances of Gln50 and Gln71 side chains and Thr59 main chain. **b)** The ratio of intensities in the paramagnetic state on the diamagnetic state are plotted (blue curve) along the S100B amino acid sequence (inset). A decrease of intensity indicates proximity to the paramagnetic probe. The yellow bars are undetected signals in ^{15}N-S100B-Ca^{2+} in the presence of reduced nitroxide labelled MTBR (diamagnetic state) due to K18 interaction.

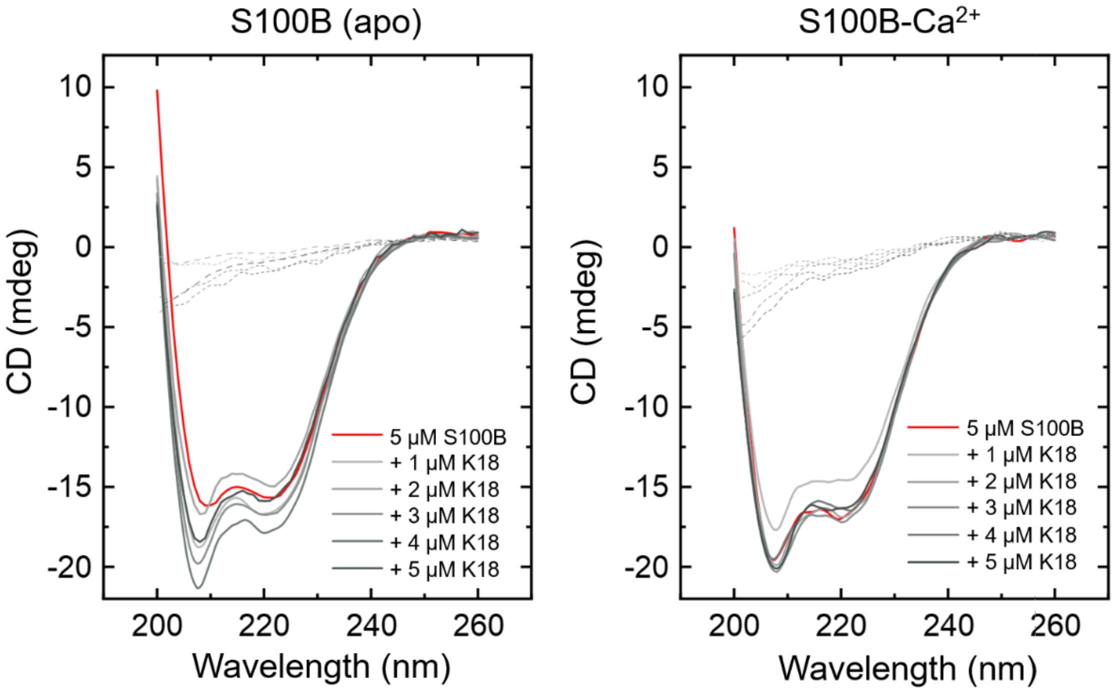

Supplementary Figure 11 - Far-UV CD analysis of interaction between S100B and K18.

5 µM S100B in the apo (left) and Ca^{2+}-bound state (right) was titrated with 1, 2, 3, 4 and 5 µM K18 and the far-UV CD spectra recorded. Measurements were made in 50 mM Tris-HCl pH 7.4, in the presence of 1.1 mM $CaCl_2$ in the assays with S100B-Ca^{2+}. Dashed traces are the spectra of K18 at the same concentrations in the absence of S100B. Traces represent averaged spectra obtained from eight accumulations for each condition.

	S100B (dimer)	Tau-K18	S100B (dimer) + Tau-K18
**Data-collection parameters**			
Beamline	B21<br>Diamond Light Source	B21<br>Diamond Light Source	B21<br>Diamond Light Source
Detector	PILATUS 2M	PILATUS 2M	PILATUS 2M
Sample to detector distance (mm)	3897	3897	3897
Column for SEC-SAXS	Superdex 200 (3.2/300)	Superdex 200 (3.2/300)	Superdex 200 (3.2/300)
Concentration of injected sample ($mg.ml^{-1}$)	13	12	12
Volume injected (µl)	35	35	35
Wavelength (Å / keV)	0.946 / 13.1	0.946 / 13.1	0.946 / 13.1
q range ($Å^{-1}$)			
Exposure time per frame (sec)	3	3	3
Temperature (K)	288	288	288
**Structural parameters**			
$I_{(0)}$ (cm^{-1}) [from $P_{(r)}$]	$0.033 \pm 2.7 \times 10^{-5}$	$0.077 \pm 1.8 \times 10^{-5}$	$0.014 \pm 2.3 \times 10^{-5}$
R_g (Å) [from $P_{(r)}$]	21.69 ± 0.025	33.73 ± 0.10	27.65 ± 0.048
$I_{(0)}$ (cm^{-1}) (from Guinier)	$0.034 \pm 3.4 \times 10^{-5}$	$0.008 \pm 2.6 \times 10^{-5}$	$0.014 \pm 2.8 \times 10^{-5}$
R_g (Å) (from Guinier)	22.41 ± 0.04	34.28 ± 0.19	28.23 ± 0.09
D_{max} (Å)	68.4	101	81.4
Porod volume estimate ($Å^3$)	26160	50500	40500
Dry volume calculated from sequence ($Å^3$)	25902	17155	43036
**Molecular-mass determination**			
Partial specific volume ($cm^3.g^{-1}$)	0.7279	0.7424	0.7337
Contrast ($\Delta\rho$ x 10^{10} cm^{-2})	2.936	2.848	2.896
Molecular mass M_r [from $I_{(0)}$] (Da)	22400 ± 100	39300 ± 250	38700 ± 200
Calculated M_r from sequence (Da)	21416	14188	35586
**Software employed**			
Primary data reduction	*ScÅtter, Chromixs*	*ScÅtter, Chromixs*	*ScÅtter, Chromixs*
Ab initio analysis	*DAMMIF, DENSS*	*DAMMIF, DENSS*	*DAMMIF, DENSS*
Validation and averaging	*DAMAVER, DENSS*	*DAMAVER, DENSS*	*DAMAVER, DENSS*
Rigid-body modelling	*Chimera*	n.a.	*Chimera*
Three-dimensional graphics representation	*Chimera*	n.a.	*Chimera*

Supplementary Table 1 – SAXS data-collection and scattering-derived parameters

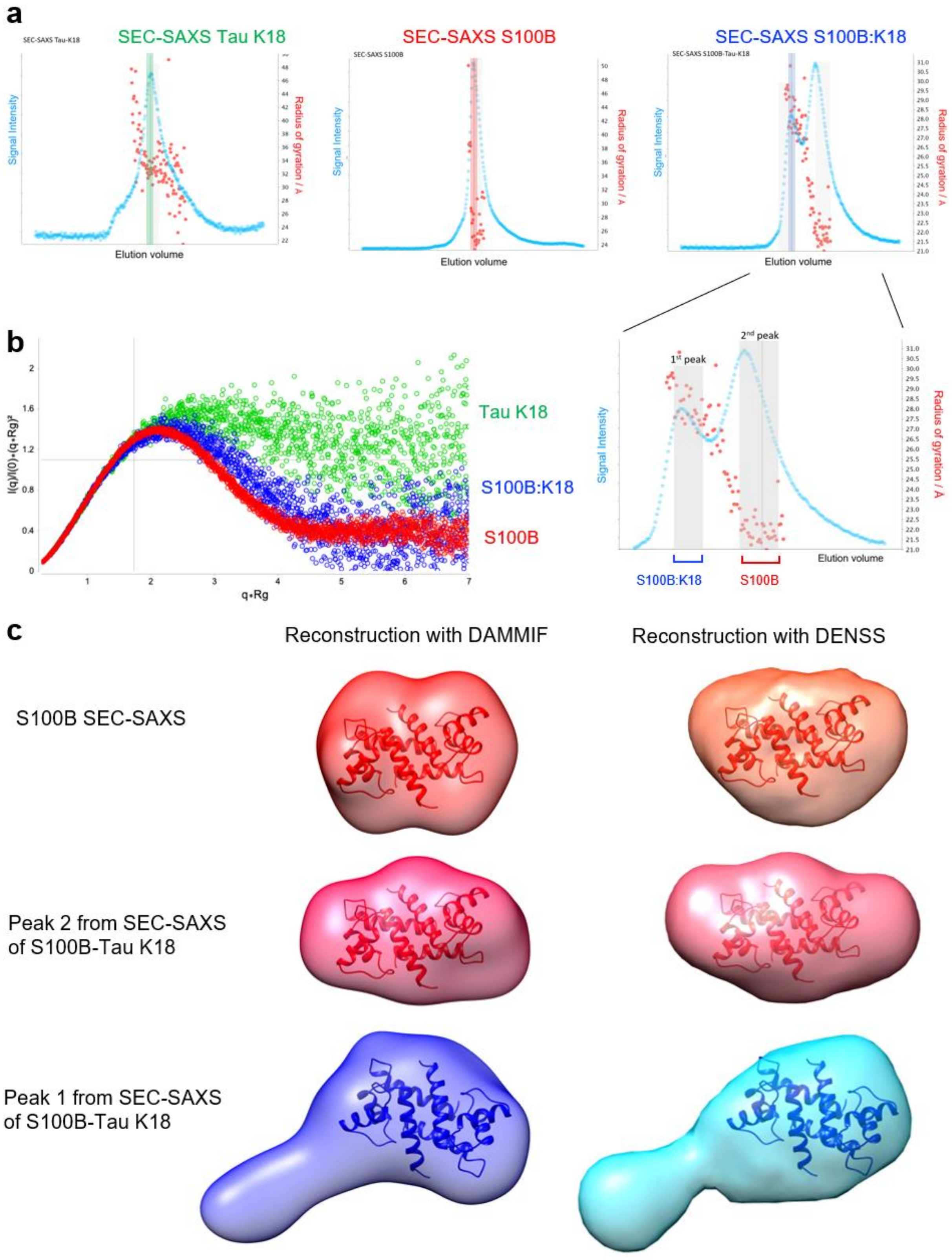

Supplementary Figure 12 - SEC-coupled SAXS data, data analysis and 3D reconstructions.

a) Size exclusion chromatography (SEC) profiles showing the intensity (blue circles) of the scattered X-rays versus the elution volume. The Radius of gyration (Rg) derived from each

single measurement is shown as red circles. The region that was used to calculate an averaged scattering curve is indicated as transparent rectangle. **b)** The dimensionless Kratky plot is used to assess protein folding and shape. The plot for S100B-Ca^{2+} homodimer (red) shows an almost symmetrical peak which is indicative for a folded globular particle. In comparison the plot for S100B-Ca^{2+}: K18 complex (blue) is not symmetrical and shifted to higher q x Rg revealing a particle that contains partially unfolded or flexible portions. The plot for -K18 shows the typical plateau for unfolded proteins and is in good agreement with previous SAXS studies on tau **c)** SEC-SAXS derived envelopes reconstructed from the scattering curves. Left hand side shows reconstruction with DAMMIF (darker colours), right hand side shows reconstruction with DENSS (brighter colours). The crystal structure of S100B shown as cartoon was fitted to the envelopes. The different reconstructions for S100B-Ca^{2+} are shown in shades of red, and the reconstructions of S100B-Ca^{2+}:K18 complex in blue. First row shows the reconstructions for S100B-Ca^{2+} from SEC of S100B-Ca^{2+} alone. Second row shows the reconstructions derived from the second peak of SEC of S100B-Ca^{2+} / K18. The SAXS envelope shows that this peak corresponds to S100B-Ca^{2+} alone. Third row shows the reconstructions derived from the first peak of SEC of S100B-Ca^{2+} / K18 alone. The SAXS envelopes are larger than those for S100B-Ca^{2+} alone and correspond to a S100B-Ca^{2+}:K18 complex. Regarding the binding stoichiometry, the 3D molecular envelope reconstructed from the SAXS suggests that one K18 is bound to the S100B dimer. This stoichiometry is derived from the major peak from a SEC-SAXS experiment. However, one cannot rule out that there may also be complexes formed by one S100B dimer and 2 K18 molecules. In the SEC-SAXS profile there is a shoulder running ahead of the major peak that harbours a S100B dimer/K18 complex. The radius of gyration from particles in this shoulder, indicates that these are larger and could arise from a (S100B dimer / 2 K18) complex. However, the resolution and signal strength in this region of the SEC-SAXS profile are rather low, precluding an accurate analysis and 3D reconstruction.

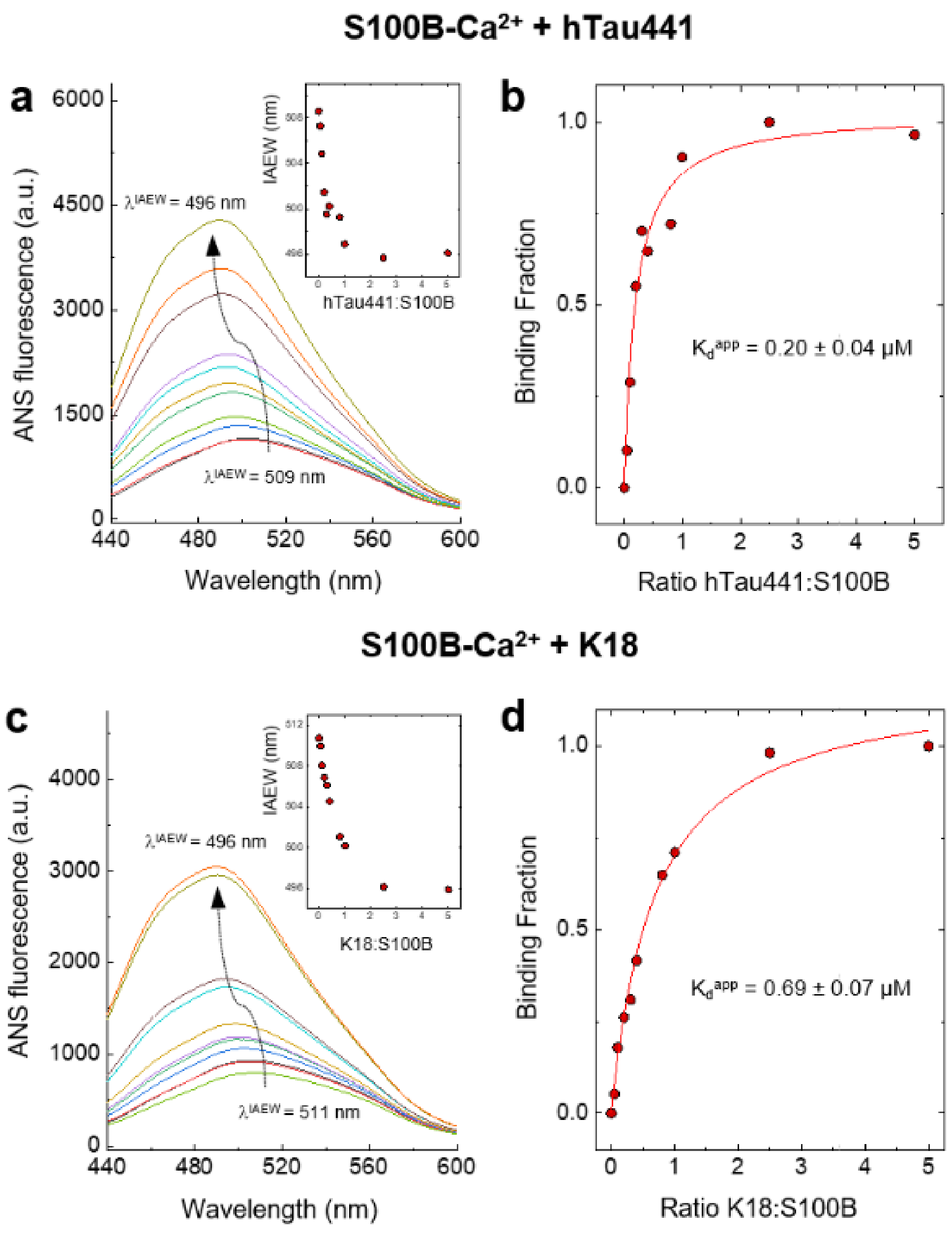

Supplementary Figure 13 - ANS-fluorescence monitored binding between S100B-Ca^{2+} and hTau441 and the K18 fragment.

a) and **c)**, 10 µM S100B in 50 mM Tris pH 7.4 and in presence of 1.1 mM $CaCl_2$ was titrated with different concentrations (0.5 - 50 µM) of hTau441 (**a**) and K18 (**c**); Insets: Intensity-averaged emission wavelength (IAEW) calculated from spectrum for each protein ratio. **b** and **d**, normalized IAEW data (binding fraction) fit estimated the apparent dissociation constant (K_d^{app}) of hTau441 (**b**, 0.20 ± 0.04 µM) and K18 (**d**, 0.69 ± 0.07 µM) from S100B. Samples in presence of 100 µM ANS were prepared and incubated at least 30 min at room temperature before measurement at 25 °C in a cuvette fluorimeter with 380 nm of excitation wavelength. Binding fraction was calculated through normalization of the IAEW and fit was performed using a one site binding model.

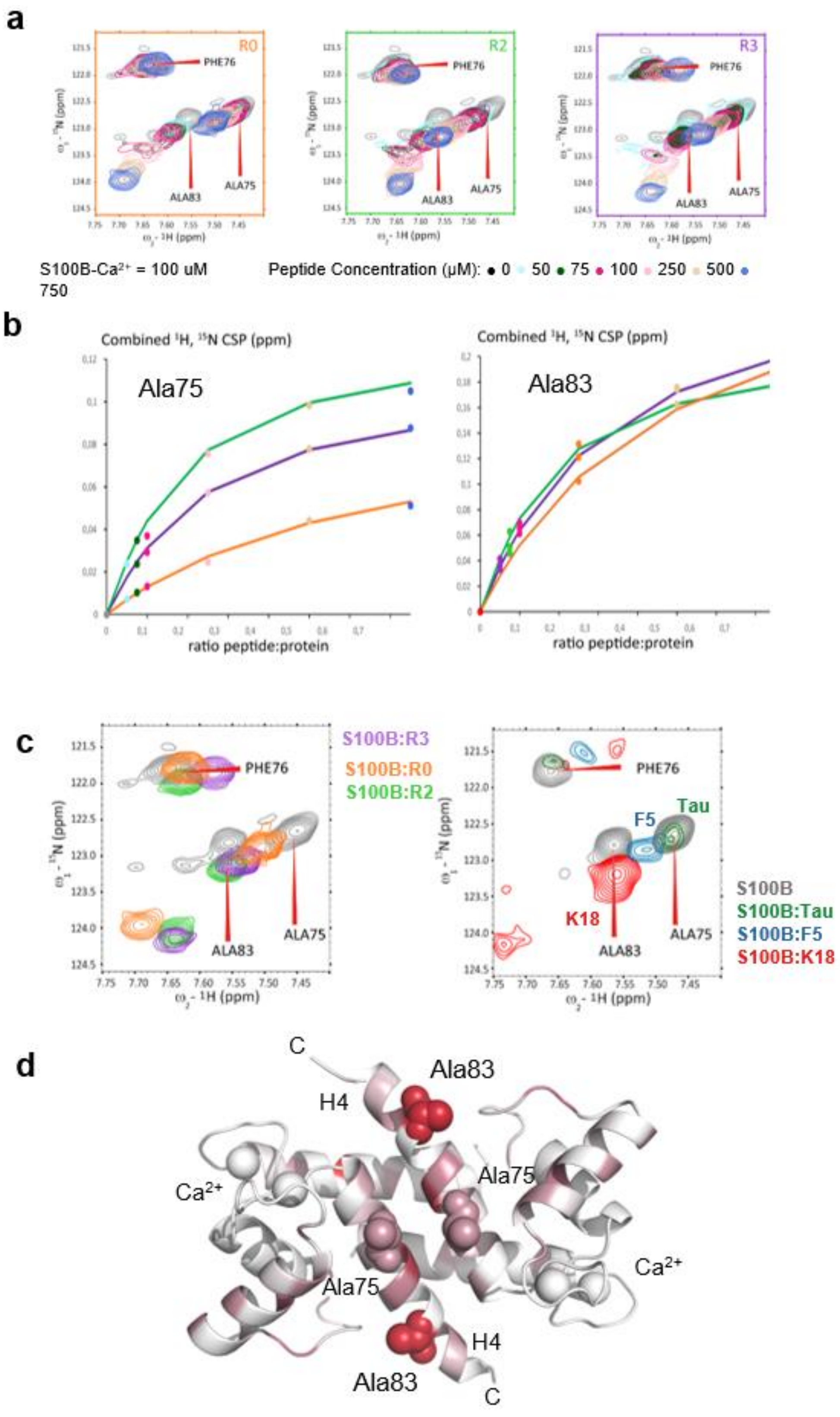

Supplementary Figure 14 - NMR monitored titration experiments of ^{15}N-S100B-Ca^{2+} with R0, R2 and R3 peptides

a) Overlayed enlarged region of ^{15}N-S100B free in solution (gray spectrum) or in the presence of 0.5 (light blue), 0.75 (green), 1 (dark pink), 2.5 (light pink), 5 (light beige) and 7.5 (blue) molar equivalents of peptides R0 (boxed in orange), R2 (boxed in green) and R3 (boxed in

purple). **b)** For each of the titration corresponding to spectra in a), combined ^{1}H, ^{15}N chemical shift variations for resonances corresponding to Ala75 (left plot) and Ala83 (right plot) are plotted for each S100B/Peptide ratio. Experimental data points are coloured coded as in a); solid lines correspond to the saturation fit (see Materials and methods), for each peptide (R0 in orange, R2 in green and R3 in purple). **c)** Overlayed enlarged region of ^{15}N-S100B free in solution (gray spectrum) or in the presence of (left spectra) 7.5 molar equivalents of peptides R0 (in orange), R2 (in green) and R3 (in purple) or (right spectra) of full-length Tau (in green, 1 S100B/1.6 Tau molar ratio), PRR (or F5 fragment, 1 S100B/1.6 F5 molar ratio, in blue), MTBR (or K18 fragment, 1 S100B/1 K18 molar ratio in red). Spectra in c) show that all resonances corresponding to a residue are being roughly localized on a so-called diagonal (sampling of the same free and bound conformation), indicative of a similar binding mode for the peptides, the fragments, and the full-length tau protein. Note that in some of the complexes with tau and its fragments, resonances are broadened. **d)** Localization on S100B structure of Ala75 and Ala83, in Helix IV (H4) that delimitates the binding cleft in the S100B dimer, colour coded according to chemical variations corresponding to the observed perturbations upon addition of peptide R2, as in Fig. 3c.

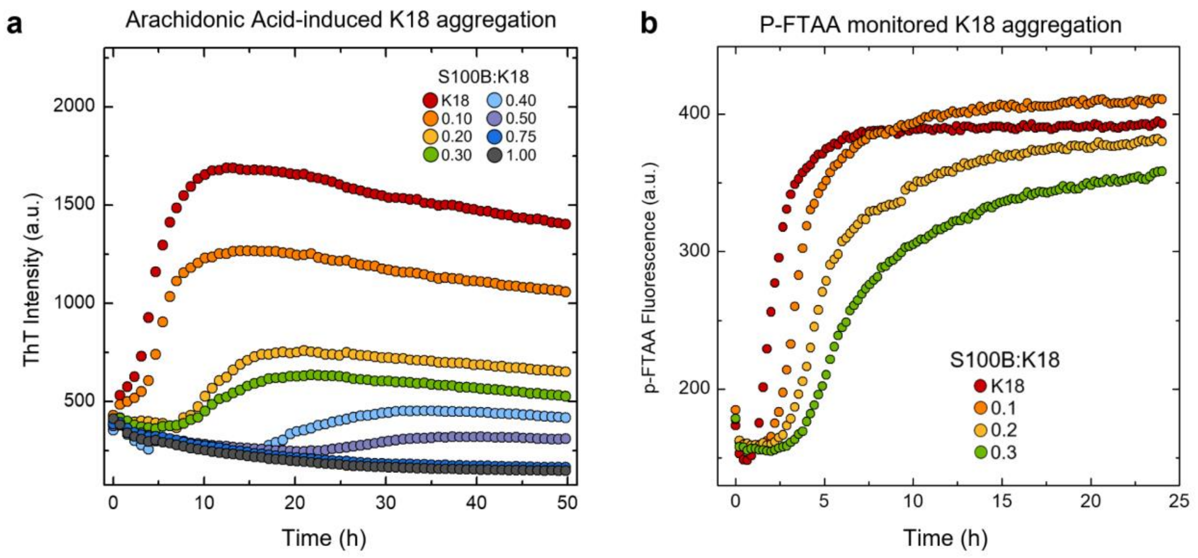

Supplementary Figure 15 - Inhibition of tau aggregation by S100B-Ca^{2+} is independent of the amyloid-detecting fluorophore and of aggregation inducer.

a) Tau aggregation followed by ThT fluorescence intensity of 10 µM K18 (red) in 50 mM Tris-HCl pH 7.4 in the presence of 1.1 mM $CaCl_2$ at 37 °C in quiescent conditions and in presence of 0.10 (orange), 0.20 (yellow), 0.30 (green), 0.40 (light blue), 0.50 (purple), 0.75 (blue) and 1.00 (grey) molar equivalents of S100B. Aggregation induced by 375 µM arachidonic acid addition. **b)** Fibril formation followed by p-FTAA fluorescence intensity of 10 µM K18 (red) in 50 mM Tris-HCl pH 7.4 in the presence of 1.1 mM $CaCl_2$ at 37 °C in quiescent conditions and in presence of 0.10 (orange), 0.20 (yellow), 0.30 (green) molar equivalents of S100B. Aggregation induced by 5 µM heparin and followed by 0.5 µM p-FTAA addition. In both panels, plots represent averaged normalized intensity curves obtained from three independent replicates for each of the tested conditions.

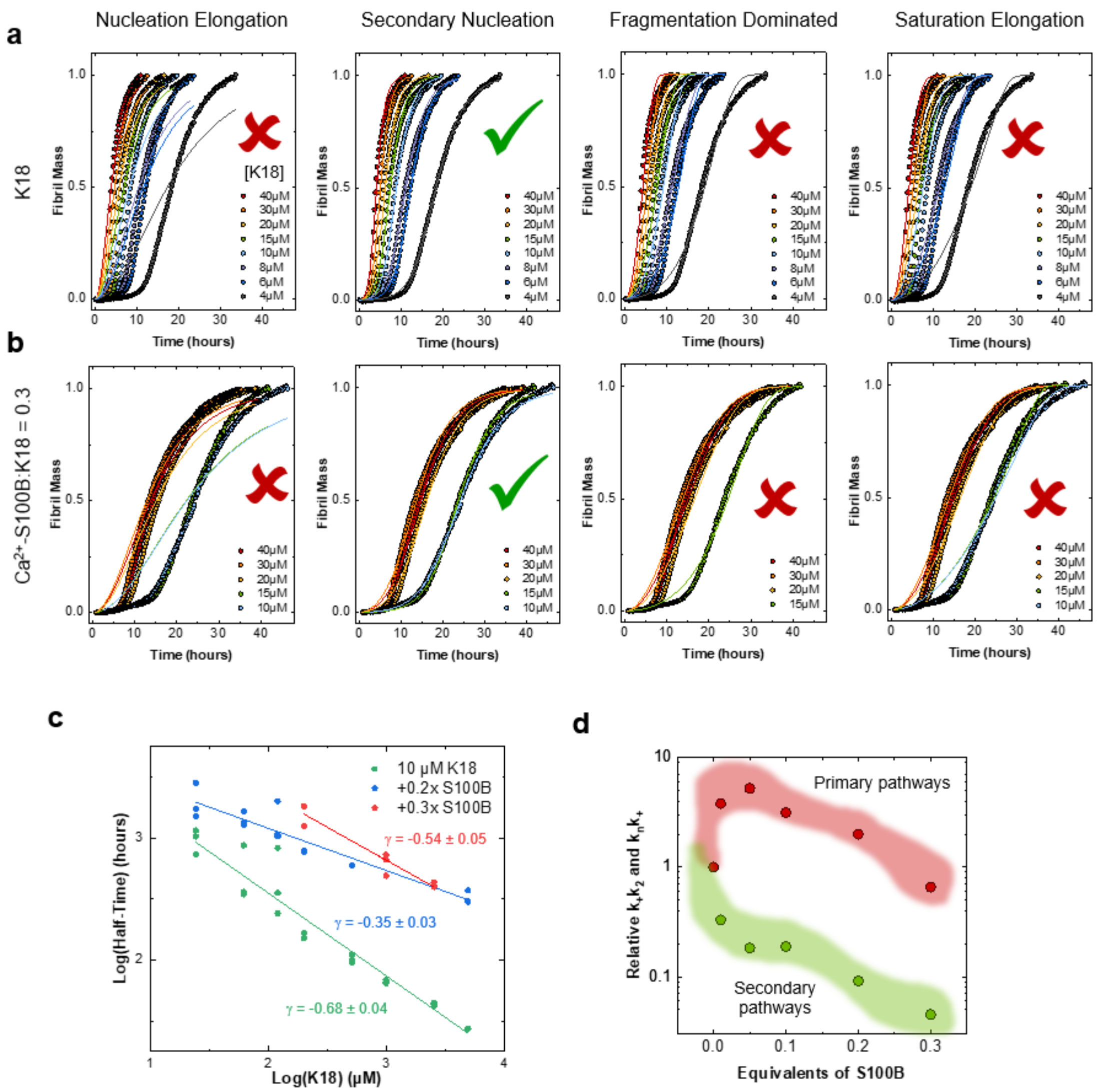

Supplementary Figure 16 - Mechanistic analysis of effect of S100B over K18 aggregation.

a-b, Fibril formation of K18 at 40 (red), 30 (orange), 20 (yellow), 15 (green), 10 (cyan), 8 (purple), 6 (blue) and 4 µM (grey) in 50 mM Tris pH 7.4 in presence of 1.1 mM $CaCl_2$ at 37 °C in absence (**a**) or presence of 0.3x molar equivalents S100B (**b**). Experimental data points were fitted using different aggregation models, available online on AmyloFit platform (from left to right: nucleation elongation, secondary nucleation, fragmentation, and saturation elongation dominated), represented by lines with the same colour code. Plots represent averaged normalized intensity curves obtained from three independent replicates for each of the tested conditions. **c**, Log-log plot of aggregation half-times for each K18 concentration. Samples were performed with K18 alone (green), in the presence of 0.2x (blue) and 0.3x (red) molar equivalents of S100B. The slope of each linear fit represents the scaling exponent for each molar ratio of S100B. **d**, relative effect of S100B on the primary nucleation (red, $k_n k_+$) and on the secondary nucleation (green, $k_+ k_2$) pathways of K18 aggregation model. Combined rate constants were obtained from secondary nucleation model fits to experimental data in Fig. 4f.

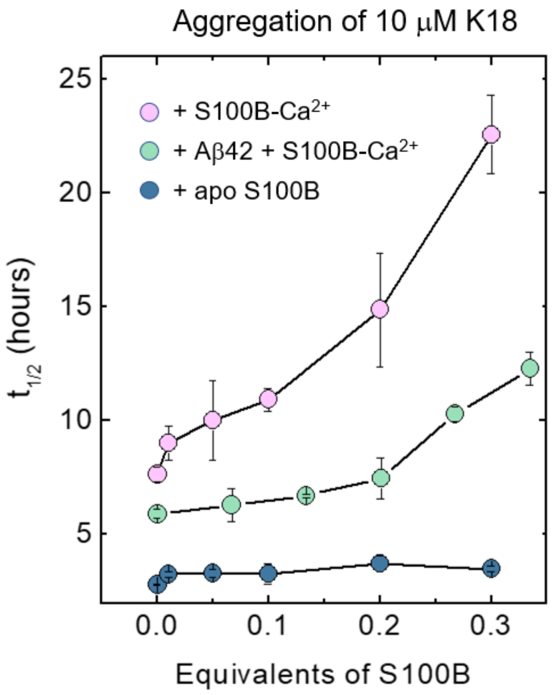

Supplementary Figure 17 - Effect of Aβ42 on the inhibition of tau-K18 aggregation by S100B.

Aggregation of 10 µM K18 was monitored in the presence of apo S100B (blue), S100B-Ca^{2+} (pink) and S100B-Ca^{2+} + 5 µM Aβ42, at different S100B equivalents in 50 mM Tris-HCl pH 7.4 (with or without 1.1 mM Ca^{2+}, as applicable) and 37 °C. At least three independent experiments were performed for each condition from which the reaction half times ($t_{1/2}$) were determined. Data are presented as mean values ± SD from n = 3 independent experiments.

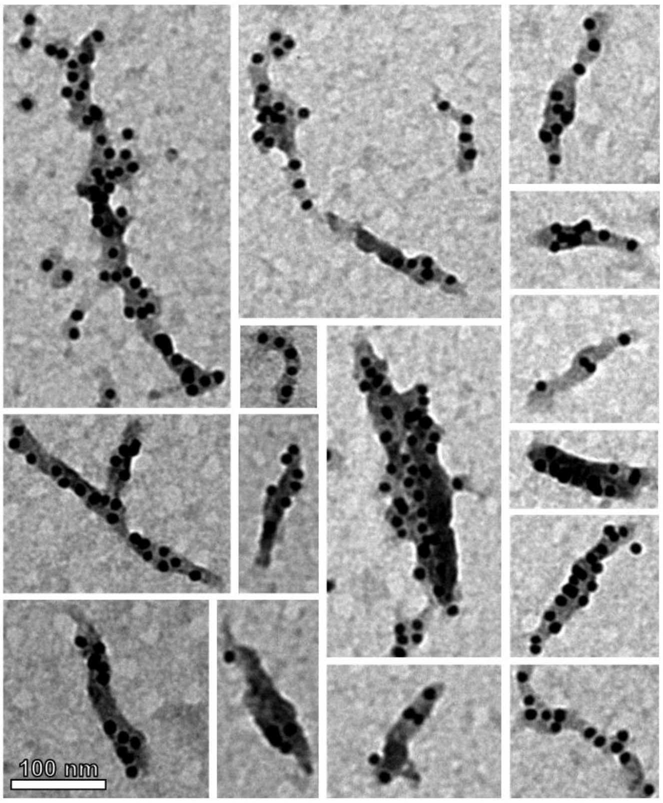

Supplementary Figure 18 - Immunogold labelling of tau fibrils with anti-S100B antibody.

Representative images of short tau (stacked) fibrils were selected to investigate if, during tau aggregation, S100B binding occurs preferentially at the extremities of fibrils (which would suggest a preferred inhibitory action over fibril elongation) or indiscriminately along the fibrils (which would suggest an effect over surface-catalysed secondary nucleation processes). Data shows that S100B (10 nm) is evenly distributed on the surface of this type of tau fibrils which stack and further evidence the labelling pattern, in agreement with predominant inhibition of secondary nucleation. Scale bar 100 nm.

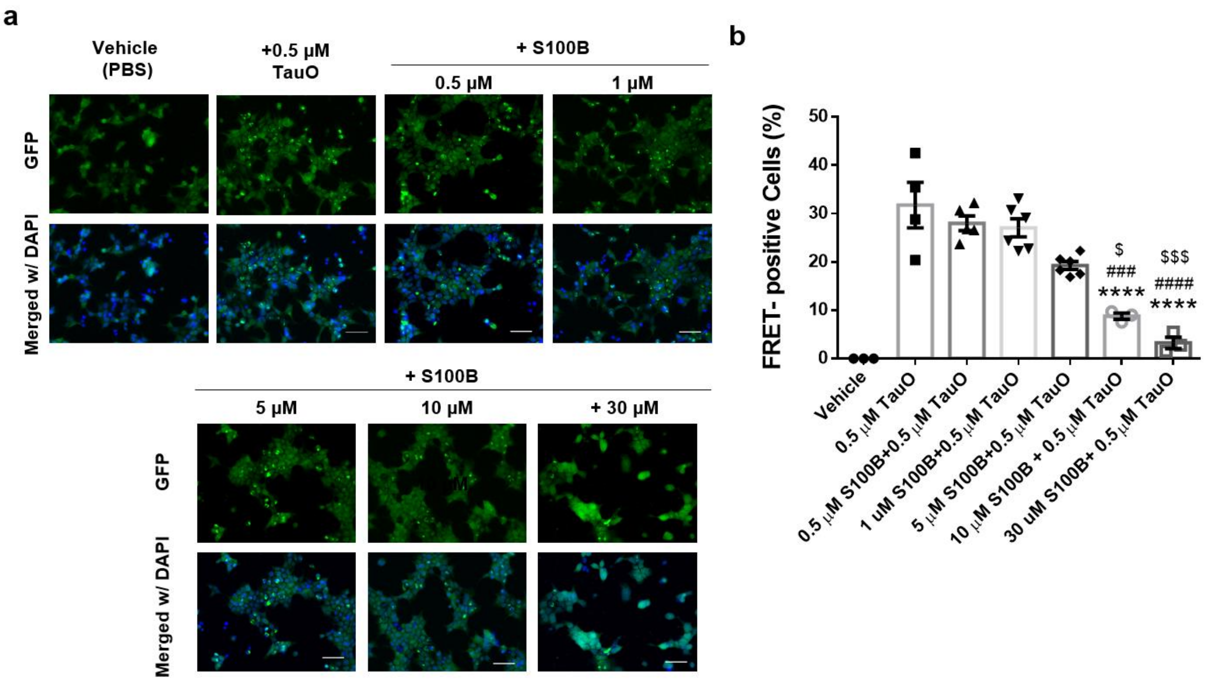

Supplementary Figure 19 - Inhibition of seeding mediated by Tau oligomers on Tau RD P301S FRET biosensor cells at different S100B levels.

a) Representative immunofluorescence images of Tau RD P301S FRET biosensor cells exposed to either Tau oligomers (0.5 µM) alone (a–c) or to Tau oligomers pre-incubated with increasing concentrations of S100B (0.5-30 µM). Cell nuclei stained with DAPI (blue). Objective used: 40X; Scale bar 50 µm. **b)** Statistical analysis of the effect of S100B (0.5-30 µM) over seeding mediated by Tau oligomers (0.5 µM). Data presented as mean and standard error of mean from n ≥ 3 independent experimental replicates using one-way analysis of variance (ANOVA) with Tukey's multiple comparison test. Statistical significance at $p < 0.05$ was considered. Statistical comparison between S100B concentrations with 0.5 µM TauO: 0.5 µM/10 µM and 0.5 µM/30 µM, ****$p < 0.0001$; 1 µM/10 µM, ### $p = 0.0001$ and 1 µM/30 µM, #### $p < 0.0001$; 5 µM/10 µM, \$ $p = 0.0369$ and 5 µM/30 µM, \$\$\$ $p = 0.0006$.
