# Supplementary material for: Dynamic interactions and Ca2+-binding modulate the holdase-type chaperone activity of S100B preventing tau aggregation and seeding
Source: Nat Commun. 2021 Nov 1;12:6292. doi: 10.1038/s41467-021-26584-2 (PMC8560819; doi:10.1038/s41467-021-26584-2)
Supplement: Supplementary file 2 — Description of Additional Supplementary Files [file 41467_2021_26584_MOESM2_ESM.pdf]

Description of Additional Supplementary Files

Supplementary Movie 1 - Mapping interactions of hTau441, K18 and R2 peptide on the S100B structure.

Description: Structural mapping on the S100B structure (PDB: 2H61) of the chemical shift perturbations observed upon interactions with hTau441, K18 and peptide R2. S100B residues and surface are colour code are color-coded by the variation of the combined ^{1}H, ^{15}N chemical shift values, from red ($\Delta\delta$ (ppm) = 0.5) to white (minimum, $\Delta\delta$ (ppm) = 0.05). Represented using Pymol.
