# Supplementary material for: Dynamic interactions and Ca2+-binding modulate the holdase-type chaperone activity of S100B preventing tau aggregation and seeding
Source: Nat Commun. 2021 Nov 1;12:6292. doi: 10.1038/s41467-021-26584-2 (PMC8560819; doi:10.1038/s41467-021-26584-2)
Supplement: Supplementary file 4 — Reporting summary [file 41467_2021_26584_MOESM4_ESM.pdf]

nature research

Corresponding author(s): Cláudio M. Gomes

Last updated by author(s): Oct 7, 2021

Reporting Summary

Nature Research wishes to improve the reproducibility of the work that we publish. This form provides structure for consistency and transparency in reporting. For further information on Nature Research policies, see our Editorial Policies and the Editorial Policy Checklist.

Statistics

For all statistical analyses, confirm that the following items are present in the figure legend, table legend, main text, or Methods section.

n/a	Confirmed	
☐	☒	The exact sample size (n) for each experimental group/condition, given as a discrete number and unit of measurement
☐	☒	A statement on whether measurements were taken from distinct samples or whether the same sample was measured repeatedly
☐	☒	The statistical test(s) used AND whether they are one- or two-sided<br>*Only common tests should be described solely by name; describe more complex techniques in the Methods section.*
☒	☐	A description of all covariates tested
☒	☐	A description of any assumptions or corrections, such as tests of normality and adjustment for multiple comparisons
☐	☒	A full description of the statistical parameters including central tendency (e.g. means) or other basic estimates (e.g. regression coefficient) AND variation (e.g. standard deviation) or associated estimates of uncertainty (e.g. confidence intervals)
☐	☒	For null hypothesis testing, the test statistic (e.g. F, t, r) with confidence intervals, effect sizes, degrees of freedom and P value noted<br>*Give P values as exact values whenever suitable.*
☒	☐	For Bayesian analysis, information on the choice of priors and Markov chain Monte Carlo settings
☒	☐	For hierarchical and complex designs, identification of the appropriate level for tests and full reporting of outcomes
☐	☒	Estimates of effect sizes (e.g. Cohen's d, Pearson's r), indicating how they were calculated

Our web collection on statistics for biologists contains articles on many of the points above.

Software and code

Policy information about availability of computer code

Data collection	NMR HSQC spectra collection: Bruker TopSpin software (version 3.6, Bruker)<br>Jasco FP-8200 software interface: Spectra Manager v2.13, Jasco<br>FLUOstar OPTIMA software interface: OPTIMA version: 2.20 R2, BMG Labtech<br>AFM interface software: Picoview 1.14.4 (Keysight Technologies)<br>For TEM imaging: Orious Sc1000 digital camera<br>Microscopes: Leica LAS X, Keyence BZ-800 Epifluorescence Microscope, Leica TCS SPE, Leica TCS SP8
Data analysis	Imaging analysis: ImageJ free software (v.2.1.0/1.53 https://imagej.nih.gov/ij). EZColocalization and Colocalization Finder plugins were used for colocalization studies, and NanoJ-SRRF 1.14 Stable plugin was used to process super-resolution imaging: Analyzer BZ-X Keyence Microscope<br>Puncta quantification: Cell profiler v4.2.1<br>NMR HSQC spectra analysis: Sparky software (version 3.6, University of California)<br>FLUOstar OPTIMA: MARS Data Analysis version 2.10 R3<br>Aggregation kinetics: Amylofit (https://www.amylofit.ch.cam.ac.uk)<br>AFM image processing: Gwyddion 2.56<br>Other data analysis and plotting: OriginPro version 2019b and Prism 6.0, GraphPad Software, Inc.; BoxPlotR online plotting tool for violin plots (http://shiny.chemgrid.org/boxplotr/)<br>SAXS analysis: ScÅtter (http://www.bioisis.net), DENSSWeb (https://denss.ccr.buffalo.edu); ATSAS v 3.0.4 software suite<br>Protein structure rendering: The PyMOL Molecular Graphics System version 2.3.3 and UCSF Chimera v1.15

For manuscripts utilizing custom algorithms or software that are central to the research but not yet described in published literature, software must be made available to editors and reviewers. We strongly encourage code deposition in a community repository (e.g. GitHub). See the Nature Research guidelines for submitting code & software for further information.

Data

Policy information about availability of data

All manuscripts must include a data availability statement. This statement should provide the following information, where applicable:

- Accession codes, unique identifiers, or web links for publicly available datasets
- A list of figures that have associated raw data
- A description of any restrictions on data availability

The NMR data generated in this study have been deposited in the Zenodo database under accession code DOI:10.5281/zenodo.5511948 [https://zenodo.org/record/5511948#.YU4ZwJrMKUI]. All other data generated in this study are provided in the Supplementary Information/Source Data file.

Field-specific reporting

Please select the one below that is the best fit for your research. If you are not sure, read the appropriate sections before making your selection.

[x] Life sciences [ ] Behavioural & social sciences [ ] Ecological, evolutionary & environmental sciences

For a reference copy of the document with all sections, see nature.com/documents/nr-reporting-summary-flat.pdf

Life sciences study design

All studies must disclose on these points even when the disclosure is negative.

Sample size	For BiFC experiments and colocalization experiments, we aimed at typical sample sizes used in the analysis of microscopy images (n=3-5, 200 cells/events per group as e.g. in https://doi.org/10.1016/j.celrep.2019.11.058. However, we also carried out a pilot experiment including 100 cells from 5 different fields for each group, to have an estimate of the average and standard deviation we could expect. Using these preliminary data, samples sizes were calculated by means of Sigmaplot v.11, aiming at a power of 0.95, alpha 0.05. Kinetic experiments were performed with at least three replicates in each condition. In BiFC experiments, the number of cells analysed was 927 (Tau/Tau) and 873 (Tau/S100B) in a total of 65 pictures/group from 4 independent experiments. In colocalization experiments, 224 cells were analysed from 3 independent experiments. For Tau RD Biosensor cells experiments no statistical methods were used to predetermine the sample size. Required sample sizes were estimated based on our experience performing similar experiments in previous publications.
Data exclusions	No data were excluded.
Replication	The data presented in the manuscript is reproducible. The interaction of Tau and S100B in Hela cells was confirmed in 5 independent experiments per each experimental group, using different batches of cells and plasmids. Colocalization experiments were repeated independently 3 times. Kinetic experiments performed over multiple of S100B concentrations were set up at least two replicates to each condition. Tau RD Biosensor cells experiments consistent results obtained from three biological replicates with four technical replicates per experiment were used in the manuscript. Independent TEM and AFM experiments of both morphological analysis and immunogold labelling were performed at least 3 times, and images presented are representative of the results obtained.
Randomization	For cellular studies results were obtained from random 4-5 regions of interest per technical replicates. For BiFC and colocalization experiments, all samples were allocated randomly into experimental groups. In colocalization experiments, the selection of the areas of interest was automated.
Blinding	For Tau RD Biosensor cells experiments investigators were blinded to group allocation during data collection and analysis. For other experiments including BiFC analysis, blinding was not possible due to limited human resources, as there was only one person to carry out the experiments, collect the samples and analyse results.

Reporting for specific materials, systems and methods

We require information from authors about some types of materials, experimental systems and methods used in many studies. Here, indicate whether each material, system or method listed is relevant to your study. If you are not sure if a list item applies to your research, read the appropriate section before selecting a response.

Materials & experimental systems

n/a	Involved in the study	
[ ]	[x]	Antibodies
[ ]	[x]	Eukaryotic cell lines
[x]	[ ]	Palaeontology and archaeology
[x]	[ ]	Animals and other organisms
[x]	[ ]	Human research participants
[x]	[ ]	Clinical data
[x]	[ ]	Dual use research of concern

Methods

n/a	Involved in the study	
[x]	[ ]	ChIP-seq
[x]	[ ]	Flow cytometry
[x]	[ ]	MRI-based neuroimaging

Antibodies

Antibodies used	Recombinant anti-S100 beta antibody [EP1576Y] monoclonal antibody from rabbit (ab52642, Abcam); anti-Tau antibody (Tau-5) monoclonal from mouse (sc-58860, Santa Cruz); anti-GAPDH antibody (0411) (sc-47724, Santa Cruz); anti-mouse IgG Alexa Fluor 647-conjugated (ref A-21235, Thermo Fisher Scientific, Waltham, MA, USA) and anti-rabbit IgG Alexa Fluor 488-conjugated (ref O-6381,Thermo Fisher Scientific, Waltham, MA, USA); goat anti-Mouse IgG (H+L) Secondary Antibody, HRP (Cat. A16066, ThermoFisher Scientific, Waltham, MA, USA); Goat anti-mouse IgG (H+L) 15nm gold-conjugated antibody (EM.GMHL15, BBI Solutions); Goat anti-rabbit IgG 10nm gold-conjugated antibody (EM.GAR10, BBI Solutions).
Validation	Recombinant Anti-S100 beta antibody [EP1576Y] monoclonal antibody from rabbit, Abcam, Cat No ab52642. Application: For detection of human, rat and mouse S100B by ICC/IF, IHC (PFA fixed), IHC-Fr, IHC-P, IP and WB. Validation/Positive controls: 1) IHC-P: Human, mouse and rat cerebral cortex; Human spiral ganglion and melanoma tissue; Normal WT and laser-treated mouse retina; Native and acellular peripheral nerve sections; Embryonic mouse brain tissue, brain tissue; 2) WB: B16F0 and A-375 cell lysates, mouse spinal cord, rat brain; 3) ICC/IF: A-375 cells. 4) IP: Human fetal brain; 5) IHC-Fr: Mouse and rat cerebrum, Hu cerebral cortex. Relevant literature reports: 1) Shi M et al. Identification of glutathione S-transferase pi as a protein involved in Parkinson disease progression. Am J Pathol 175:54-65 (2009). WB ; Human.<br><br>Tau (TAU-5) is recommended for detection of both phosphorylated and nonphosphorylated Tau proteins of mouse, rat, human and bovine origin by Western Blotting (starting dilution to be determined by researcher, dilution range 1:10-1:200), immunoprecipitation [1-2 µg per 100-500 µg of total protein (1 ml of cell lysate)], immunofluorescence (starting dilution to be determined by researcher, dilution range 1:10-1:200) and immunohistochemistry (including paraffin-embedded sections) (starting dilution to be determined by researcher, dilution range 1:10-1:200); non cross-reactive with Tubulin or other microtubule associated proteins. Positive Controls: SK-N-SH cell lysate: sc-2410, mouse brain extract: sc-2253 or TE671 cell lysate: sc-2416. (https://datasheets.scbt.com/sc-58860.pdf).<br><br>GAPDH (0411) from santa cruz (sc-47724) is recommended for detection of GAPDH of human origin by Western Blotting (starting dilution 1:200, dilution range 1:100-1:1000), immunoprecipitation [1-2 µg per 100-500 µg of total protein (1 ml of cell lysate)], immunofluorescence (starting dilution 1:50, dilution range 1:50- 1:500) and immunohistochemistry (including paraffin-embedded sections) (starting dilution 1:50, dilution range 1:50-1:500); not recommended for detection of GAPDH of mouse or rat origin. Positive Controls: HeLa whole cell lysate: sc-2200, Jurkat whole cell lysate: sc-2204 or K-562 whole cell lysate: sc-2203. (https://datasheets.scbt.com/sc-47724.pdf)<br><br>More information at the manufacturer's website.

Eukaryotic cell lines

Policy information about cell lines

Cell line source(s)	HeLa human cervix adenocarcinoma cells (ATCC, CRM-CCL-2)<br>Tau RD P301S CFP/YFP FRET Biosensor cells (ATCC, CRL-3275)<br>SH-SY5Y human neuroblastoma cells (ATCC ,CRL-2266)<br>U-251 MG human glioblastoma cells (ECACC, 08061901)
Authentication	HeLa cells were authenticated by frequent check of the morphology by optical microscopy at high and low culture densities. The cell line was a low-passage cell line acquired from ATCC. We did not pass our batches more than 20 times and never let them over-cultured.<br>Tau RD Biosensor cells were authenticated by the supplier.
Mycoplasma contamination	Cell lines tested negative to mycoplasma contamination using the MycoSpy kit (Biontex Laboratories GmbH)
Commonly misidentified lines (See ICLAC register)	No commonly misidentified lines were used in this study.
